# Signaling pathways in cancer-associated fibroblasts and targeted therapy for cancer

**DOI:** 10.1038/s41392-021-00641-0

**Published:** 2021-06-10

**Authors:** Fanglong Wu, Jin Yang, Junjiang Liu, Ye Wang, Jingtian Mu, Qingxiang Zeng, Shuzhi Deng, Hongmei Zhou

**Affiliations:** State Key Laboratory of Oral Diseases, National Center of Stomatology, National Clinical Research Center for Oral Diseases, West China Hospital of Stomatology, Sichuan University, Chengdu, Sichuan People’s Republic of China

**Keywords:** Drug development, Cancer microenvironment

## Abstract

To flourish, cancers greatly depend on their surrounding tumor microenvironment (TME), and cancer-associated fibroblasts (CAFs) in TME are critical for cancer occurrence and progression because of their versatile roles in extracellular matrix remodeling, maintenance of stemness, blood vessel formation, modulation of tumor metabolism, immune response, and promotion of cancer cell proliferation, migration, invasion, and therapeutic resistance. CAFs are highly heterogeneous stromal cells and their crosstalk with cancer cells is mediated by a complex and intricate signaling network consisting of transforming growth factor-beta, phosphoinositide 3-kinase/AKT/mammalian target of rapamycin, mitogen-activated protein kinase, Wnt, Janus kinase/signal transducers and activators of transcription, epidermal growth factor receptor, Hippo, and nuclear factor kappa-light-chain-enhancer of activated B cells, etc., signaling pathways. These signals in CAFs exhibit their own special characteristics during the cancer progression and have the potential to be targeted for anticancer therapy. Therefore, a comprehensive understanding of these signaling cascades in interactions between cancer cells and CAFs is necessary to fully realize the pivotal roles of CAFs in cancers. Herein, in this review, we will summarize the enormous amounts of findings on the signals mediating crosstalk of CAFs with cancer cells and its related targets or trials. Further, we hypothesize three potential targeting strategies, including, namely, epithelial–mesenchymal common targets, sequential target perturbation, and crosstalk-directed signaling targets, paving the way for CAF-directed or host cell-directed antitumor therapy.

## Introduction

Cancer, as a major public health problem worldwide, is the second leading cause of death with an estimated 10.0 million globally in 2020.^1,2^ Majority of cancer deaths from cancers are caused by local recurrence and/or distant organ/tissue metastasis.^3,4^ If the cancers are identified in the early stage and occur in the original lesion site, the total 5-year relative survival rate of the ten most common cancers is ~34.2–100%, with a local recurrence rate of <16.1% after surgery, radiation, and/or chemotherapy, while the total 5-year relative survival rate drops to 2.5–30.2% for advanced cancers with frequent recurrences and/or metastasis,^5,6^ thereby requiring more aggressive treatments, including immunotherapy, biological therapy, or targeted therapy, etc. However, the recurrent or metastatic cancers can exhibit quick progression and/or become resistant to therapeutic strategies, which are exclusively or mainly aimed at cancer cells. One of the main reasons for the failure of cancer treatment is that the tumor microenvironment (TME) is fully or partially ignored in the development of antitumor therapy.^7^ Since cancer progression is highly associated with the physiological state of TME, targeting nonneoplastic stromal components that substantially contribute to tumor progression are considered for cancer treatment.^8^

Cancer-associated fibroblasts (CAFs), as the major components of TME, have been extensively explored and are known to be involved in diverse cellular processes, including cell differentiation, proliferation, and stemness; extracellular matrix (ECM) remodeling; and cell migration and apoptosis, all of which can exert critical roles in tumor biological behaviors, including tumorigenesis, tumor growth, energy metabolism, tumor immunity, angiogenesis, tumor progression, recurrence, and metastasis.^9–11^ The biological activities of CAFs are mediated by various intracellular and extracellular factors, especially those in signaling pathways closely related to cancer progression, which might be targeted for anticancer therapy. Since CAFs exert molecular and functional heterogeneity in different cancers and even in different stages of the same type of tumor and because of the specific crosstalk between CAFs and cancer cells,^12^ any therapeutic strategies developed should exploit the specificity and diversity of CAFs to optimize treatment efficacy for targeted therapy. To better understand the nature of CAFs, herein, we summarize historic milestones of the basic research and clinical studies on CAFs, especially those focused on precursors of CAFs and CAFs isolation, heterogeneity, signaling pathways, and involvement in cancer therapy and therapy resistance, and suggest new potential therapeutic strategies.

## Definition of fibroblasts

Fibroblasts were firstly identified in the 1850s as spindle-shaped cells in connective tissue that can synthesize collagen^13^ and originated mostly from the primitive mesenchyme of the mesoderm,^14^ and, in some cases, partially from the neural crest of the ectoderm.^15,16^ Indeed, the definition of fibroblasts is constantly changing. It is widely accepted that fibroblasts in normal tissues are generally embedded within fibrillar ECM as single resting mesenchymal cells.^17^ They are also defined by their cell morphology, tissue location, and lack of the lineage markers that are expressed by other cells, such as epithelial cells and leukocytes.^15^ Currently, vimentin is widely used to compare fibroblasts with cells expressing epithelial markers, and fibroblast‐specific protein 1 (FSP1, also known as S100A4) is considered as a reliable marker for quiescent fibroblasts.^18^ However, vimentin and FSP1 are also expressed by cells in mesenchymal lineages in addition to fibroblasts; thus, cellular shape and location are frequently combined for the identification of fibroblasts,^19–23^ demonstrating that details on the lineage of fibroblasts remain to be determined.

Quiescent fibroblasts in the interstitial space are the major producers of ECM under normal physiological conditions and can be reversibly activated to facilitate repair and regeneration in response to tissue damage.^24^ Preceding their functioning in the regeneration stage, quiescent fibroblasts are activated into myofibroblasts and then accumulate at the sites of repair for wound healing.^25^ In these cases, activated fibroblasts secrete transforming growth factor-beta (TGF-β) and acquire a contractile phenotype via the expression of α-smooth muscle actin (α-SMA), thereby effectively closing wounds.^26^ In addition, fibroblasts are also critical for the homeostasis of adjacent epithelial cells, acting in an indirect paracrine manner, similar to that of growth factors^27,28^ or via direct mesenchymal–epithelial cell interactions.^29,30^ In angiogenesis with increased production of vascular endothelial growth factor A (VEGFA),^31^ the immune response, and keratinocyte proliferation, fibroblasts play roles by secreting cytokines and chemokines.^27,32^ Further, ECM development mediated by fibroblasts in lymph nodes acts as a “highway” to transport potential antigens and contributes to the migration of leukocytes,^33^ indicating that the structural roles of fibroblasts allow effective immune responses. Interestingly, when wounds heal, activated fibroblasts are restored to the quiescent phenotype owing to apoptosis,^34^ indicating that reversibility is a hallmark feature of fibroblasts associated with tissue repair.

## From fibroblasts to CAFs in TME

### Cellular sources and heterogeneity of CAFs

**Fig. 1:**
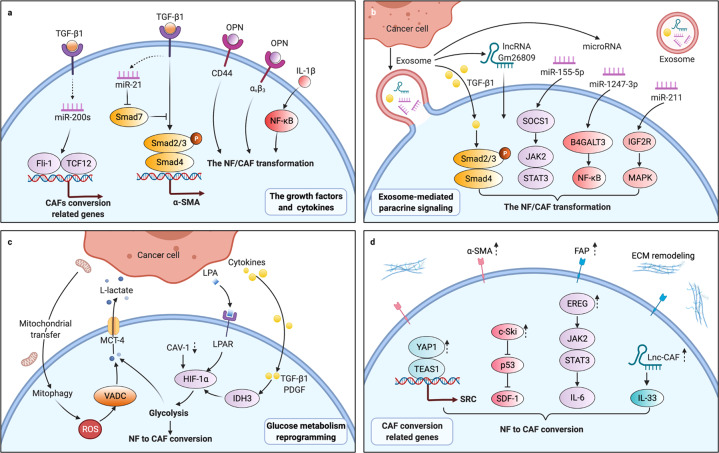
Conversion from normal fibroblasts (NFs) to cancer-associated fibroblasts (CAFs). **a** Grow factors and cytokines such as transforming growth factor-beta 1 (TGF-β1), osteopontin (OPN), and IL-1β combined with their reporters in NFs, then activated the downstream effector including miRNAs and CD44, etc. to regulate the targeted gene expression of CAFs through TGF-β/Smads and nuclear factor kappa-light-chain-enhancer of activated B cells (NF-κB) signaling pathways. **b** Cancer-derived exosomes shuttling cargos such as miRNAs and lncRNAs transformed NFs to CAFs via the downstream signals including TGF-β/Smads, Janus kinase/signal transducers and activators of transcription (JAK/STAT), NF-κB and mitogen-activated protein kinase (MAPK) cascades. **c** NF-CAF conversion was driven by glucose metabolism reprogramming and hypoxia-inducible factor-1α (HIF-1α) signaling pathway was implicated in this glycolysis. **d** Changes in cellular homeostasis triggered the self-propelled conversion by regulating the cytoskeletal proteins activation and secreted phenotype through the JAK/STAT and p53 signaling pathways. Fli-1 leukemia integration 1, TCF12 transcription factor 12, SOCS1 suppressor of cytokine signaling 1, B4GALT3 β-1,4-galactosyltransferases III, IGF2R insulin-like growth factor 2 receptor, LPA lysophosphatidic acid, VDAC voltage-dependent anion channel, YAP1 Yes-associated protein 1, TEAD1 TEA domain transcription factor-1, SDF-1 stromal cell-derived factor-1, also known as CXCL12, EREG epiregulin, ROS reactive oxygen species

Cancers, as ongoing and unabated injurious stimuli, initiate fibroblasts irreversibility transition, driving acquisition of cancer-associated phenotypes (Fig. 1). The irreversibility transitions could be driven in a variety of ways. First, TME as a reservoir of growth factors, cytokines, and other factors signals to resident fibroblasts contributing to the transformation of normal fibroblasts (NFs) to CAFs. A diverse set of factors, including TGF-β1, osteopontin (OPN), and interleukin-1β (IL-1β), etc., which are released from cancer cells and/or immune cells,^35^ induce the transition of stromal fibroblasts to CAFs by regulating the TGF-β and nuclear factor kappa-light-chain-enhancer of activated B cells (NF-κB) signaling pathways.^36–39^ Then, exosomes also play essential roles in cellular communications, promoting fibroblasts to acquire new receptors or even genetic material from the cancer cells. Cancer-derived exosomes shuttling cargos such as microRNAs (miRNAs), long noncoding RNA (lncRNA) Gm26809, or TGF-β1 to reprogram NFs into CAFs via the downstream mitogen-activated protein kinase (MAPK), NF-κB, signal transducers and activators of transcription 3 (STAT3), or TGF-β signaling cascades.^38,40–42^ Also, a shift in energy metabolism such as aerobic glycolysis is potentially considered as a priming event in the conversion of NFs into CAFs. Lysophosphatidic acid (LPA), TGF-β1, or platelet-derived growth factor (PDGF) from cancer cells are able to induce aerobic glycolysis of fibroblasts via hypoxia-inducible factor-1α (HIF-1α) pathway; fibroblasts can also be metabolically reprogrammed via caveolin-1 (CAV-1) downregulation or cancer cell-derived mitochondrial transfer.^43–46^ In addition, various evidence has proven that the conversion of NFs into CAFs is accompanied by changes in the self-expression of certain components. For instance, Yes-associated protein 1 (YAP1) in NFs, as a transcriptional coactivator, modulates the transcription of SRC by forming a protein compound with TEA domain transcription factor-1 (TEAD1), resulting in cytoskeletal protein activation and ultimately transformation into CAFs.^47^ Overall, induced by cancer cells and TME, etc., NFs are activated to CAFs. Compared to resting NFs, CAFs acquire enhanced proliferative and secretory capabilities, which contribute to ECM remodeling, autocrine activation, and immunomodulatory function. Activated CAFs are characterized by different markers that are expressed at low levels or not expressed in NFs (Table 1). Among them, all or part of a combination with α-SMA, fibroblast-activated protein (FAP), and PDGF receptor α/β (PDGFRα/β) could be used to distinguish CAFs from NFs in cancers. Some markers, since CAFs are suggested to represent a heterogeneous population of cells,^48^ are required to characterize this heterogeneity.

**Table 1:** Phenotype and heterogeneity of CAF

Tissue type	Phenotype	Markers	Origin and/or function	Ref.
BC (human)	CAF-S1	CD29^Med^, FAP^Hi^, FSP1^Med^, α-SMA^Hi^, PDGFRβ^Med-Hi^, CAV-1^Low^	Regulatory of cancer invasion and immune response	^58^
	CAF-S2	CD29^Low^, FAP^Neg^, FSP1^Neg-Low^, α-SMA^Neg^, PDGFRβ^Neg^, CAV-1^Neg^	ND	
	CAF-S3	CD29^Med^, FAP^Neg^, FSP1^Med-Hi^, α-SMA^Neg-Low^, PDGFRβ^Med^, CAV-1^Neg-Low^	ND	
	CAF-S4	CD29^Hi^, FAP^Neg^, FSP1^Low-Med^, α-SMA^Hi^, PDGFRβ^Low-Med^, CAV-1^Low^	Regulatory of actin cytoskeleton and oxidative metabolism	
OSCC (human)	CAF-N	KGF	High fibroblast motility	^60^
	CAF-D	TGF-β1	Low fibroblast motility	
	NF	HGF, MMP3	Lower tumor incidence	
LC (human)	Cluster 1	*COL10A1*	Showing a strong EMT signals	^65^
	Cluster 2	COX4I2, *ACTA2*, *MEF2C*	Regulatory of myogenesis and angiogenesis	
	Cluster 3	ND	Upregulating collagen and ECM molecules expression	
	Cluster 4	*PLA2G2A*	Similar to cluster 1	
	Cluster 5	*MMP3*	Low myogenesis and high mTOR expression	
	Cluster 6	FIGF	Representing nonmalignant fibroblasts	
	Cluster 7	ND	Similar to cluster 4 but differing in the glycolysis pathway	
CRC (human)	CAF-A	*MMP2*, *DCN*, *COL1A2*	Regulatory of ECM remodeling and express FAP	^66^
	CAF-B	*ACTA2*, *PDGFA*, *TAGLN*	Activation of cytoskeletal gene	
	NF	*MGP* (ND)	ND	
HNSCC (human)	CAF1	*CTHRC1, COL1A1, POSTN, TPM4, MFAP2* (ND)	Promoting cancer metastasis	^67^
	CAF2	*CFD, APOD, CXCL12, GPC3, SEPP1* (ND)		
	NF	Depleted of markers for myofibroblasts and CAFs	Resting fibroblasts	
OC (human)	FAP-high CAF	FAP, TGF-β, COL11A1, SULF1, IL-6, CXCL12	Regulatory of cancer invasion and immune regulation	^345^
	FAP-low CAF	DLK1, TCF21, COLEC11	Regulatory of glucose homeostasis, lipid metabolism, etc.	
	NF	COMP, SFRP2, GJB2 (ND)	ND	
BC (mouse)	mCAF	Fibulin-1, PDGFRα	From resident fibroblast/regulatory of tumor immune response	^57^
	vCAF	Nidogen-2	From vasculature/promoting vascular development	
	cCAF	Ki-67	Representing the proliferative segment of vCAF	
	dCAF	SCRG1	From malignant cell/locating on tumor–stroma boundary	
PDAC (mouse)	myCAF	α-SMA	Adjacent to tumor cells and promoting desmoplasia	^61^
	iCAF	IL-6, LIF	Locating away within stroma and promotes tumor progression	
	NF	ND	Pancreatic stellate cells	
BC (mouse)	Cluster 0	*Ly6c1*	From resident fibroblasts/promoting cancer progression and immune evasion	^68^
	Cluster 1	α-SMA	Promoting cancer development and progression	
	Cluster 2	*Cdk1*	Identifying as dividing cells	
	Cluster 3	*Cd53*	High transcriptional enrichment for desmin	
	Cluster 4	*Crabp1*	From Ly6c1^high^ fibroblasts	
	Cluster 5	*Cd74*	Expressing MHC class II and regulatory of immune-modulatory	
HCC (ND)	Activated myofibroblast phenotype	α-SMA, FAP, vimentin, vollagen 1α, PDGFRα, FN	Maintaining and enhancing the stemness of HCC cells	^398^
	Mesenchymal stromal cell phenotype	CD90, CD73, CD105, CD29, CD44, CD166	Regulatory of immunosuppression	

*α-SMA* alpha-smooth muscle actin, *ACTA2* actin alpha 2, *APOD* apolipoprotein D, *BC* breast cancer, *CAF* cancer-associated fibroblast, *CD* clusters of differentiation, *CAV-1* caveolin-1, *Cdk1* cyclin-dependent kinases 1, *CFD* complement factor D, *COL1* collagen type I, *COLEC11* human collectin subfamily member 11, *COX4I2* cytochrome *c* oxidase subunit 4I 2, *Crabp1* cellular retinol-binding protein-I, *CRC* colon adenocarcinoma, *CTHRC1* collagen triple helix repeat-containing protein 1, *CXCL12* C–X–C motif chemokine 12, *DCN* decorin, *DLK1* delta-like 1, *ECM* extracellular matrix, *ELK3* ETS-domain protein, *EMT* epithelial–mesenchymal transition, *FAP* fibroblast activation protein, *FIGF* c-fos-induced growth factor, *FN* fibronectin, *FOXO1* forkhead box protein O1, *FSP1* fibroblast activation protein 1, *GPC3* glypican-3, *HCC* hepatocellular carcinoma, *HNSCC* head and neck squamous cell carcinoma, *HOXB2* homeobox 2, *IL* interleukin, *KGF* keratinocyte growth factor, *LC* lung cancer, *LIF* leukemia inhibitory factor, *MEF2C* myocyte enhancer factor 2C, *MFAP2* microfibrillar associated protein 2, *MMP* matrix metalloproteinase, *ND* not determined, *OC* ovarian cancer, *OSCC* oral squamous cell carcinoma, *PDAC* pancreatic ductal adenocarcinoma, *PDGF* platelet-derived growth factor, *PLA2G2A* phospholipase A2 group IIA, *POSTN* periostin, *SEPP1* selenoprotein P1, *SCRG1* scrapie responsive protein 1, *SULF1* sulfatase1, *TAGLN* transgelin, *TCF21* transcription factor 21, *TGF* transforming growth factor, *TPM4* tropomyosin-4

It is becoming clear that there are subpopulations of CAFs for distinct functional states, raising the question of what determines the CAFs’ heterogeneity. Overwhelming evidence suggests that CAFs’ heterogeneity includes different organs/tissues, sources, functions, secretion types, and others.^49,50^ The alterations in CAFs show a remarkable spectrum of organs/tissues specificity. For example, CAV-1 was found to induce glycometabolic reprogramming in breast CAFs,^51^ while CAV-1-induced aerobic glycolysis was not completely verifiable in oral CAFs.^52^ Therefore, some alterations in CAFs appear only in cancers from one or a few tissue types, instead of a pan-cancer genome and transcriptome commonalities. The heterogeneity of CAFs in the same organ or tissue is likely held to depend on their precursor fibroblasts.^53^ Generally, CAFs are derived from the activated local tissue-resident fibroblasts, fibrocytes recruited from bone marrow, mesenchymal stem cells (MSCs) and stellate cells, or are the products of the mesenchymal transition of epithelial and endothelial cells, and the transdifferentiation of pericytes, smooth muscle cells, and adipocytes.^54,55^ Depending on their origin, the functions, and markers of CAF subtypes are diverse and unique. The CAF subtypes from local tissue-resident fibroblasts are similar to myofibroblasts with high expression of cytoskeletal proteins like α-SMA for cell contraction, while the CAF subtypes are derived from perivascular cells might be associated with metastasis. However, drawing definitive conclusions on the cellular origins of CAFs is difficult because currently there is no available means to track the conversion between cell states directly or to collect longitudinal samples from the same lesion in human tissue. Mouse models with well-characterized disease progression have been created to shed light on the origin of CAFs.^56^ In a mouse model of breast cancer, three transcriptionally diverse subpopulations of CAFs were defined via various lineage sources.^57^ In addition, the cues emanating from molecular phenotypes or secretion phenotypes might also determine the CAFs’ heterogeneity. Recently, single-cell RNA-sequencing and conventional RNA-sequencing of human tissues have allowed better unbiased assessment of heterogeneous CAFs.^20,58,59^ By analyzing a combination of classical markers, such as FAP and PDGFRβ, CAFs in breast cancer were distinguished by levels of marker expression.^58^ Another classic way to identify CAFs involves analyzing the different secretory phenotypes exhibited in different subtypes. For instance, elastin and collagen levels are distinctively expressed in CAFs of the lung TME.^60^

Accordingly, the high heterogeneity in CAFs raises an interesting question: If CAFs would switch in distinct functional states or subtypes? As an answer to this question, it has been suggested that IL-1 signaling induces the generation of inflammatory CAFs, and TGF-β antagonizes CAF switching from an inflammatory phenotype to a myofibroblast phenotype.^61,62^ Taken together, all these evidences show that the discovery of the heterogeneity of CAFs revealed a remarkably complex and diverse portrait.

### Methods for isolation and culture of CAFs

Progression in heterogeneity studies requires more accurate methods for isolation and culture of CAFs. Without question, fibroblasts are easily isolated and cultured on plastic, e.g., human skin, mouse ears, and tail tips can be used as sources to isolate fibroblasts that can be digested and cultured in a medium.^63^ Using the typical curettage method combined with trypsinization or enzyme digestion methods for CAF primary cell culture, this model is unnecessary to purify cells prior to culture because of their rapid initial proliferation of fibroblasts. Antibiotics and additional washing steps are usually included in the culture process to prevent infections with bacterial and/or mycoplasma. Epithelial cells growing either in groups or scattered among the CAFs can be easily removed because of differences in adhesion ability and tolerance to trypsin of these two cell types, greatly contributing to further research on CAFs.^64^ In 2006, our group separated the CAFs from human oral cancer tissues using this curettage method.^22^ However, in these studies involving CAFs, caveats were included to suggest subtle variations in various subtypes requiring the need for new markers. CAF subtypes can be identified through multicolor flow cytometry (fluorescence-activated cell sorting). After tissue digestion, lineage markers are used to exclude hematopoietic, epithelial, and endothelial cells, and various combinations of CAF markers are used for CAF subtype identification.^58^ CAF subtypes can also be identified through single-cell transcriptomics and mass cytometry methods.^57,65–68^ Although the α-SMA and FAP staining for distinction CAFs from NFs are available, putative CAF subtype identification methods still require more reproducibility, validation, and repeated optimization.

In primary cell cultures, early passaged and immortalized CAFs have functions that can be directly investigated in vitro, and it is crucial to replicate the TME considering the intricate interactions among tumor cells, CAFs, and other stromal cells. The crosstalk of CAFs with cancer cells has been evaluated through various culture patterns. Cells can be directly cocultured and indirectly cocultured in Transwell chambers or conditioned medium (CM).^69,70^ Indeed, we extended the two-dimensional (2D) culture of fibroblasts from oral precancerous lesions with the addition of *Candida.*^71^ Furthermore, techniques differ for cell culture in 2D and 3D, with the latter allowing patterns of growth in vitro to better mimic that of the tissue architecture in vivo.^72^ One type of 3D coculture is implemented through the use of reconstituted matrices. The solid porous scaffold is based on a range of natural and synthetic materials and serves as a membrane providing a platform that can be added to a mixture of different cells, including CAFs. For instance, this scaffold‐based technology can be used to replicate tissue architecture, which is composed of alternate layers of cells, and especially for tumors of the epithelium with CAFs.^73,74^ Aggregate culture platforms of particular interest are scaffold‐free systems, also referred to as spheroids or organoids, in which heterogeneous populations can be evaluated for drug resistance and sensitivity or can be used to establish hypoxic cancer models.^72,75,76^ However, organoids commonly contained only epithelial cells and lack fibroblasts and types of other cells, such as immune and endothelial cells.^77^ To overcome these limitations of organoids, a multilayer bladder called an “assembloids” has been created by reconstituting tissue stem cells with stromal components representative of an organized architecture.^78^ In sum, either classical 2D/3D cultures or assembloids will benefit functional studies of CAFs in the context of the gradually accepted importance of TME.

## Major signaling pathways and targeted therapies in CAFs

Many signaling pathways have been explored extensively in CAF-mediated cancer progression for their roles in carcinogenesis, tumor growth, cell migration and invasion, energy metabolism, and cancer recurrence and metastasis. Various endogenous and exogenous factors in CAFs, including biomarkers, cytokines, chemokines, miRNAs, and lncRNAs, are involved in the regulation of these signaling pathways. Several major signal cascades affect not only the biological behaviors of CAFs themselves but also the crosstalk between CAFs and cancer cells. Therefore, in this section, we will discuss how signaling pathways regulate the CAFs, the crosstalk of CAFs with cancer cells, and the targeted therapies.

### TGF-β signaling pathway

#### TGF-β signaling pathway in CAFs and its targeted therapy

Were it not for the fibroblasts, TGF-β would more likely be discovered many years later, because TGF-β was initially identified by its ability to stimulate the growth of rat fibroblasts.^79^ In the canonical TGF-β signaling pathway, one group of TGF-β superfamily ligands, including the TGF-β/Activin/Nodal, bind to TGF-β type II receptor (TGF-βRII), which phosphorylates TGF-βRI. The binding of TGF-βRII and TGF-βRI propagates signaling by phosphorylating Smad2/3, while Smad1/5/8/9 are mediated by another group of TGF-β ligands, such as bone morphogenetic protein (BMP), through binding of BMP-RII and BMP-RI. Phosphorylated Samd2/3 heterotrimerize with Smad4 and translocate into the nucleus as a transcription factor complex, subsequently regulating the transcription of TGF-β target genes. Inhibitory Samd6/7 binds to activated type I receptors and then inhibit signal transduction of the TGF-β family.^80,81^ In the noncanonical TGF-β signaling pathway, TGF-β superfamily ligands can activate Rho, extracellular signal-regulated kinase (ERK), Janus kinase/STAT3, and phosphoinositide 3-kinase (PI3K)/AKT pathways in CAFs.^51,82,83^

Over the past nearly four decades, TGF-β has been further explored and found to be widely produced by nearly all cell types including CAFs, and the TGF-β signaling pathway has been found to have pleiotropic effects on CAF behaviors through autocrine and paracrine mechanisms.^15,38,84^ Resident NFs can be induced to transition into CAFs by TGF-β1 in various tumors, including bladder, breast, colorectal, and pancreatic cancer,^38,85,86^ indicating that TGF-β1-driven CAF generation is a common event during cancer development. Mechanistically, TGF-β1 alters the target gene expression of stromal fibroblasts through the canonical TGF-β signaling pathway, leading to differential gene expressions such as α-SMA and FAP in CAFs.^37,87^ After treatment with TGF-β1, MSCs were induced to differentiate into CAFs through the activation of the JAK/STAT3 signaling cascade, and inhibition of TGF-β/Smads signaling pathway reduced the transformation.^84,88,89^ These data suggest that both canonical and noncanonical TGF-β pathways exhibit roles in promoting CAFs generation. In addition, CAF proliferation was attenuated by a TGF-β receptor inhibitor (LY2109761) in hepatocellular carcinoma (HCC),^90^ and CAF migration was enhanced by TGF-β1 through overexpression of the tight junction protein occludin in colon cancer.^83^ Paracrine TGF-β caused the activation of noncanonical TGF-β/RhoA/ROCK axis signaling, as well as the TGF-β canonical pathway that induced transcriptional regulation of Snail1 and Twist1 target genes to increase CAFs contractility and ECM remodeling.^82^ Of note, since the cellular biological behaviors are driven by energy, as a hallmark of cancer, metabolic reprogramming of CAFs is defined as reverse Warburg effect (RWE), characterized by increased lactate, glutamine, nucleotides, fatty acids, and pyruvate derived from aerobic glycolysis.^51,91^ Recently, studies have supported the supposition that TGF-β signaling pathway plays a critical role in RWE mainly through metabolic reprogramming-related proteins, including CAV-1 and isocitrate dehydrogenase-3α (IDH3α).^92,93^ Mechanistically, CAV-1 interacted with the TGF-βRI, and induced its degradation, and then suppressed TGF-β-dependent Smad2 phosphorylation and nuclear translocation.^94^ TGF-β overexpression in CAFs decreased mitochondrial activity and increased glycolysis via CAV-1 downregulation in breast cancer, and TGF-β1-induced CAFs switched metabolic programming from oxidative phosphorylation to aerobic glycolysis by downregulating IDH3α in colon cancer.^92,93^ Importantly, CAF-specific endoglin (TGF-β family coreceptor) targeted by a neutralizing antibody (TRC105) decreased the metastatic spread of colorectal cancer cells to the liver in vivo.^37^ Summary, not only the components of canonical and noncanonical TGF-β signaling pathways in CAFs could be targeted for antitumor therapy but also the biomarkers such as CAF-derived CAV-1 and endoglin, etc., have great potential to be targeted in cancer treatment.

#### TGF-β signaling pathway-mediated crosstalk of CAFs with cancer cells and its targeted therapy

The CAF-mediated TGF-β pathway contributes to cancer progression by regulating many physiological processes, including cancer cell proliferation, migration, invasion, and metastasis.^95^ Previous studies showed that TGF-β-activated CAFs secreted growth factors, including TGF-β, fibroblast growth factor 2/7 (FGF2/7), VEGF, PDGF, and hepatocyte growth factor (HGF), to promote cancer cell proliferation.^96^ CAFs stimulated gastric cancer cell migration and invasion, which were attenuated by Smad2 small interfering RNA (siRNA) and anti-TGF-β-neutralizing antibody.^97^ TGF-β-activated ECM remodeling in CAFs created biochemical and mechanical stimuli for the invasion of cancer cells.^92,98–100^ Intriguingly, HCC cells were found to have high levels of connective tissue growth factor (CTGF) as a consequence of elevated TGF-β1 expression, and LY2109761 (a TGF-β receptor inhibitor) not only suppressed CAF proliferation but also alleviated CTGF expression, thereby reducing tumor growth and dissemination,^90^ indicating that TGF-βR-targeted therapy seems to have good efficacy in terms of antitumor metastasis. Consistently, CAFs contributed to the “education” of cancer cells, changing their behavior from indolent or nonaggressive into that of an invasive and metastatic phenotype.^101,102^ However, in our preliminary studies and the data from Wang lab, TGF-βRI mutation was detected in ~19% of head and neck squamous cell carcinoma (HNSCC) with metastasis and decreased or abrogated TGF-βRII/TGF-βRIII expression was evident in 35.3% of human oral squamous cell carcinoma (OSCC) on the protein level and in >70% of human HNSCC at the messenger RNA levels.^103–105^ These data have illustrated that TGF-βR-targeted therapy exerts strict indications, and its mutation needs to be detected before targeting TGF-βR. Notably, the nutrients recycled through the RWE via CAF-derived CAV-1 could be transferred into adjacent tumor cells to promote cancer progression in a paracrine fashion,^94^ while loss of CAV-1 in the tumor–stroma led to activated TGF-β signaling to trigger the epithelial–mesenchymal transition (EMT) of cancer cells,^92,106,107^ demonstrating that CAF-derived CAV-1 plays a paradoxical role in tumor progression, and any targeted strategies used to exploit the dual roles of CAV-1 in TGF-β signaling pathway should be developed with consideration of the ability of CAV-1 to transition from acting as a tumor promotor to acting as a suppressor to optimize treatment efficacy.

**Fig. 2:**
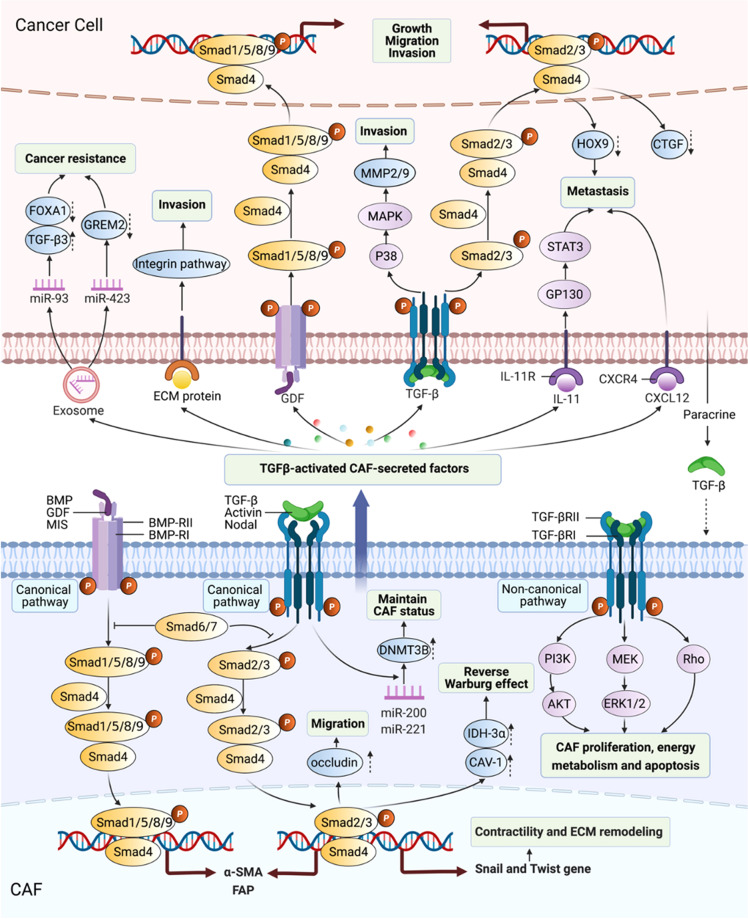
TGF-β signaling pathway-mediated crosstalk between CAFs and cancer cells. The canonical TGF-β signaling pathways consist of TGF-β/Activin/Nodal-Smads pathway and bone morphogenetic protein/growth differentiation factor/Müllerian-inhibiting substance (BMP/GDF/MIS)-Smads pathway. Noncanonical pathways represent those that activate TGF-βR, but induce no-Smads pathway. Within tumor microenvironment (TME), a large number of TGF-β protein secreted by cancer cells mediated the transformation of NFs into CAFs supporting cancer progression by activation TGF-β signaling pathway, particularly canonical pathway. Activated CAFs can be orchestrated by TME to maintain their status and promote their proliferation and migration. In turn, these adaptations would also be contributed to the formation of tumor-promoting TME. CAF-secreted factors regulated extracellular matrix (ECM) remodeling to accelerate cancer invasion and metastasis indirectly. On the other hand, most factors derived from CAFs can directly mediate intricate regulation on the cancer cells. Most proteins, such as ECM proteins, GDF, TGF-β, IL-11, and CXCL12, could activate the pathway of cancer cells to exert biological functions, including promoting cancer growth, migration, invasion, and metastasis, through receptor–ligand binding. Genetic information would also transfer from CAFs into cancer cells by extracellular vehicles. CAF-derived exosomal miR-93 and miR-423 would be endocytosed by cancer cells and then promoted cancer chemoresistance and radioresistance. FOXA1 forkhead box protein A1, MMP2 matrix metalloproteinase 2, GP130 glycoprotein 130, CTGF connective tissue growth factor, IDH3α isocitrate dehydrogenase-3α, CAV-1 caveolin-1

Indeed, the effect of autocrine TGF-β signaling on CAFs remained unclear until a study demonstrated that the establishment of TGF-β autocrine signaling pathways induced CAF formation during breast cancer progression,^108^ indicating that tumor-derived TGF-β, in a positive-feedback loop, could affect the biological characteristics of CAFs and that the crosstalk between CAFs and cancer cells is not unidirectional but bidirectional (Fig. 2). For instance, tumor-derived TGF-β was likely to recruit CAFs affiliated with the invasive front and at the bone metastatic disease to promote tumor development.^102^ Cancer cell-induced reactive oxygen species (ROS) promoted the loss of CAV-1 in CAFs via autophagy and then activated HIF-1α under ROS-induced pseudohypoxic conditions.^45,109^ In ovarian cancer through TGF-β signaling pathway, tumor-derived lysophosphatidic acid and exosomes promoted the differentiation of MSCs to CAFs,^17,102,110–113^ and cancer-derived TGF-β stimulated the expression of IL-6, C–X-C motif chemokine 12 (CXCL12), and VEGFA in CAFs to induce metastasis.^114^ Accumulating evidence suggests that abundant miRNAs in CAFs have regulatory roles in tumor progression (Table 2); for instance, targeting miR-101 attenuated TGF-β signal transduction by downregulating TGF-βR1 and Smad2 in HCC cells to suppress vascular mimicry (VM) formation.^115^ In fact, since the bidirectional crosstalk between the CAFs and cancer cells, any therapeutic strategy targeted CAFs or cancer cells might not obtain optimize efficacy. Thus, using a systems biology strategy, we combined experimental and computational analyses for the prediction of epithelial targets in an interactive network of proteins and found that TGF-βRIII would be targeted as an epithelial–mesenchymal common target (EMCT) in OSCC.^116^ In summary, the paracrine and autocrine TGF-β signaling pathway contributes extensively to the crosstalk of CAFs and cancer cells, and EMCTs show great potential for use in targeted therapy, while more studies are still needed to determine which TGF-β signaling component can serve as a common target in antitumor therapy.

**Table 2:** miRNAs in CAF

miRNAs (expression)	Effects on cancer cells and mechanism of action	Potential targeting therapy	Ref.
Lung cancer			
miR-1 (↓)	Proliferation, chemoresistance by NF-κB and Bcl-xl pathway^a^	Pathway	^399^
miR-101 (↓)	Growth, metastasis by CXCL12 and PI3K-Akt pathway^a^	Restoring miR-101	^400^
miR-210 (↑)	Migration, proliferation, invasion, EMT by PI3K/AKT pathway^a^	Exosomal mRr-210	^401^
miR-1/206 (↓)	Angiogenesis, TAMs accumulation, growth, metastasis by FOXO3a/VEGF/CCL2 signaling	Delivery of pre-miR-1/206 and anti-miR-31	^402^
miR-31 (↑)			
Breast cancer			
miR-1-3p (↓)	Progression, metastasis by *GLIS1* gene	MiR-1-3p EVs	^403^
miR‐22 (↑)	Chemoresistance by PI3K‐AKT pathway	Nanoparticles	^404^
miR-26b (↓)	Migration, invasion via TNKS1BP1/CPSF7/COL12A1^a^	MiR-26b	^405^
miR-29b (↓)	Growth, chemoresistance, migration by p38-STAT1 pathway	Suppressor miR-29b	^354^
miR-92 (↑)	PD-L1; migration, proliferation by LATS2 of Hippo pathway	MiR-92 inhibitor	^406^
miR-200b/c (↓)	Growth, active mobility, invasion by NF-κB pathway	Pathway	^407^
miR-200s (↓)	Invasion, metastasis via transcription factors Fli-1 and TCF12	MiR-200s	^408^
miR-205 (↓)	Angiogenesis by targeting YAP1 through STAT3 pathway	Pathway	^409^
miR-221 (↑)	Growth, migratory by A20/c-Rel/CTGF signaling^a^	LNA-i-miR-221	^272^
miR-320 (↓)	Proliferation, invasion by PI3K/AKT pathway	MiR-320 agents	^410^
miR-181d-5p (↑)	EMT via transcription factor CDX2 and HOXA5^a^	Exosomal miR-181d-5p	^411^
miR-3613-3p (↑)	Proliferation, metastasis by SOCS2 gene expression^a^	Exosomal miR-3613-3p	^412^
miR-4516 (↓)	Proliferation by targeting *FOSL1* gene^a^	MiR-4516 agents	^413^
miR-16/148a (↑)	Migration, metastasis by FAK pathway	Pathway	^414^
miR-141 (↓)	MiR-200b/c/miR-221/DNMT3B feedback loop influencing TGF-β1 expression, and TGF-β1/DNMT3B/miR-141 axis enhancing TCF12 in CAF to promote cancer cell proliferation^a^	Regulatory loop/axis	^415^
miR-221 (↑)			
miR-200b/c (↓)			
Prostate cancer			
miR-15/16 (↓)	Proliferation and capability of CAF by FGF2 and FGFR1	Restoring miR-15/16	^416^
miR-146a-5p (↓)	Metastasis, invasion by EGFR/ERK pathway	Exosomal miR-146a-5p	^417^
miR-409 (↑)	Tumorigenesis, EMT, and stemness by tumor suppressor genes	MiR-409	^418^
miR-423-5p (↑)	Chemoresistance by the TGF-β signaling pathway	MiR-423-5p inhibitor	^419^
Colorectal cancer			
miR-21 (↑)	Motility and invasion by MMP inhibitor RECK^a^	MiR-21	^420^
miR-31 (↑)	Radiosensitivity via genes *Beclin-1*, *ATG*, *DRAM*, and *LC3*^a^	MiR-31	^421^
miR-92a-3p (↑)	Stemness, EMT, metastasis, and chemoresistance by activating Wnt/β-catenin pathway and inhibiting mitochondrial apoptosis	Inhibiting exosomal miR-92a-3p	^422^
miR-93-5p (↑)	Radioresistance by TGF-β signaling pathway	Exosomal miR-93-5p	^367^
miR-17/192 (↓) miR-200c (↓)	Invasion by regulating ECM target genes on the protein level^a^	Restoring miR-17/192, and/or miR-200c	^423^
Gastric cancer			
miR-34 (↓)	Proliferation and invasion by targeting 16 genes	Exosomal miR-34	^424^
miR-106b (↑)	Migration and invasion by PTEN-mediated signaling pathway^a^	MiR-106b	^425^
miR-139 (↓)	Growth and metastasis by downregulating MMP11	Exosomal miR-139	^308^
miR-149 (↓)	EMT and stem-like properties by COX-2/PGE2 signaling	MiR-149	^426^
miR-214 (↓)	Migration and invasion by EMT and targeting FGF9^a^	MiR-214/FGF9	^427^
miR-522 (↑)	Suppressing ferroptosis by targeting ALOX15 and blocking lipid-ROS accumulation, chemotoxicity promoting miR-522 secretion by activating USP7/hnRNPA1 pathway	Blocking miR-522 packaging into exosomes	^9^
Hepatocellular carcinoma			
miR-29b (↓)	Invasion, migration, and apoptosis by DNMT3b^a^	MiR-29b mimic	^428^
miR-101 (↓)	Vascular mimicry formation by SDF-1 signaling	Signaling networks	^115^
miR-320a (↓)	Proliferation, migration, and metastasis by MAPK pathway	Transfer of miR-320a	^429^
miR-1247-3p (↑)	Stemness, EMT, chemoresistance, and tumorigenicity by IL-6/8; lung metastasis by β1-integrin-NF-κB pathway	Tumor–stromal crosstalk	^430^
Cholangiocarcinoma			
miR-15a (↓)	Migration by regulating PAI-2 expression^a^	MiR-15a/PAI-2 axis	^431^
Cervical and squamous cell carcinoma			
miR-10a-5p (↑)	Angiogenesis and tumorigenicity by Hedgehog pathway	MiR-10a-5p evs	^432^
Pancreatic cancer			
miR-21(↑)	Desmoplasia, drug resistance, and CAF activation by *PDCD4* gene	MiR-21	^433^
miR-106b (↑)	Chemoresistance by directly targeting *TP53INP1* gene^a^	Exosomal miR-106b	^309^
miR-146a (↑)	Proliferation and survival by gemcitabine-induced Snail pathway	Exosomal inhibitors	^70^
Head and neck cancer			
miR-7 (↑)	Proliferation and migration via RASSF2 and decreasing PAR-4^a^	Inactivation of the RASSF2-PAR-4 axis	^434^
miR-34a-5p (↓)	Proliferation and metastasis by AKT/GSK3β/β-catenin pathway	MiR-34a-5p/AXL axis	^136^
miR-196a (↑)	Proliferation and resistance by regulating CDKN1B and ING5^a^	Exosomal miR-196a	^435^
miR-3188 (↓)	Proliferation and apoptosis by targeting BCL-2	Exosomal miR-3188	^436^
Melanoma			
miR-155 (↑)	Angiogenesis by SOCS1/JAK2/STAT3 signaling pathway	Exosomal miR-155	^437^
Osteosarcoma			
miR-1228 (↑)	Migration and invasion by endogenous SCAI mRNA and protein^a^	Exosomal miR-1228	^438^
Ovarian cancer			
miR-21 (↑)	Motility, invasion, lowering chemosensitivity and apoptosis by binding to APAF1 coding sequence	Inhibiting miR-21	^439^
miR-98-5p (↑)	Cisplatin resistance by downregulating CDKN1A	Exosomal miR-98-5p	^440^
miR-31/214 (↓)	Recruitment and growth by regulating CCL5	MiRNAs	^441^
miR-155 (↑)			
Endometrial cancer			
miR-31 (↓)	Motility and invasion by targeting partially the SATB2 homeobox gene	MiR-31/SATB2 signal	^442^
miR-148a (↓)	Invasion by decreasing WNT10B in WNT/β-catenin pathway^a^	Restoring miR-148a	^443^
miR-148b (↓)	EMT by relieving the suppression of gene *DNMT1*	Transfer of miR-148b	^444^

*ALOX15* arachidonate lipoxygenase 15, *AKT* protein kinase B, *APAF1* apoptotic protease-activating factor-1, *Bcl-xL* B cell lymphoma-extra large, *CAF* cancer-associated fibroblasts, *CCL* C–C chemokine ligand, *CDKN* cyclin-dependent kinase inhibitor, *CDX2* caudal-related homeobox 2, *COX-2* cyclooxygenase-2, *CTGF* connective tissue growth factor, *CXCL* C–X–C chemokine ligand, *DNMT* DNA methyltransferase, *EGFR* epidermal growth factor receptor, *ECM* extracellular matrix, *EMT* epithelial–mesenchymal transition, *ERK* extracellular signal-related kinase, *EV* extracellular vesicles, *FAK* focal adhesion kinase, *FGF* fibroblast growth factors, *Fli-1* friend leukemia integration 1, *FOXO3a* Forkhead box O3, *GLIS1* Gli-similar 1, *GSK* glycogen synthase kinase, *hnRNPA1* heterogeneous nuclear ribonucleoprotein A1, *HOXA5* homeobox A5, *IL* interleukin, *ING5* inhibitor of growth 5, *JAK* Janus kinase, *MAPK* mitogen-activated protein kinases, *MMP* matrix metalloproteinases, *NF-κB* nuclear factor kappa-B, *PAI-2* plasminogen activator inhibitor 2, *PGE2* prostaglandin E2, *PTEN* phosphate and tensin homolog, *PI3K* phosphatidylinositol-3-kinase, *ROS* reactive oxygen species, *SCAI* suppressor of cancer cell invasion, *SDF-1* stromal cell-derived factor-1, *SOCS* suppressor of cytokine signaling, *STAT* signal transducer and activator of transcription, *TAMs* tumor-associated macrophages, *TCF12* transcription factor 12, *TGF* transforming growth factor, *USP7* ubiquitin-specific protease 7, *VEGF* vascular endothelial-derived growth factor

^a^Without xenograft models

### PI3K/AKT/mTOR signaling pathway

#### PI3K/AKT/mTOR signaling pathway in CAFs and its targeted therapy

PI3K, as an intracellular phosphatidylinositol kinase, encompasses p85 and p110.^117^ Transmembrane growth factor receptors include epidermal growth factor receptor (EGFR), G-protein-coupled estrogen receptor (GPER), VEGF, and insulin growth factor receptor 1, etc. and can activate PI3K and then phosphorylate phosphatidylinositol-4,5-bisphosphate (PIP2) to phosphatidylinositol-3,4,5-trisphosphate (PIP3).^118,119^ Then, PIP3 binds to the phosphoinositide-dependent kinase 1 (PDK1) and PDK2, and subsequently recruits AKT to the plasma membrane and phosphorylates the threonine/serine (Thr308/Ser473) phosphorylation site to activate AKT.^117–119^ Activated AKT can phosphorylate and activate its substrate mammalian target of rapamycin (mTOR) via direct and indirect pathways.^120–122^ Phosphatase and tensin homolog (PTEN), as a tumor suppressor that dephosphorylates PIP3 into PIP2 for inactivation of AKT and PDK1, negatively regulating the PI3K/AKT/mTOR signaling pathway.^117,123^

**Fig. 3:**
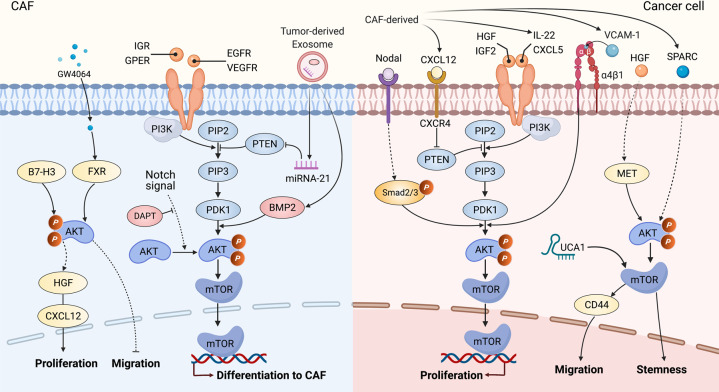
PI3K/AKT/mTOR signaling pathway in CAFs and the crosstalk of CAFs with cancer cells. In the CAFs, by the receptor–ligand binding, activated phosphatidylinositol-3-kinase (PI3K) can phosphorylate phosphatidylinositol-4,5-bisphosphate (PIP2) to phosphatidylinositol-3,4,5-trisphosphate (PIP3), while miRNA-21 could attenuate the inhibition of phosphatase and tensin homolog (PTEN) on PIP3. As a result, PIP3 activated phosphoinositide-dependent kinase 1 (PDK1)/AKT signaling cascade to transfer the rapamycin target protein (mTOR) into the nuclei, subsequently regulating the expression of targeted genes associated with differentiation into CAFs and motility, etc. Notch signaling pathway was also involved in CAF differentiation via AKT signaling pathway. B7-H3 promoted AKT phosphorylation for proliferation in CAFs, while AKT phosphorylation was involved in the inhibitory effects on the migration mediated by GW4064. Similarly, CAF-derived HGF, IGF-2, IL-22, and CXCL5 can activate PI3K/AKT/mTOR signaling axis, while CXCL12 can inhibit PTEN. Nodal-induced activation of Smad2/3 could activate AKT phosphorylation and lncRNA UCA1 collaborated with mTOR. Consequently, CAF-mediated PI3K/AKT signaling pathway regulated the cell proliferation, migration, and stemness in cancer cells. FXR farnesoid X receptor, HGF hepatocyte growth factor, IGR insulin growth factor receptor, GPER G-protein-coupled estrogen receptor, EGFR epidermal growth factor receptor, VEGFR vascular endothelial growth factor, DAPT, N-[N-(3,5-difluorophenacetyl)-l-alanyl]-S-phenylglycine *t*-butyl ester, VCAM-1 vascular cell adhesion molecule-1, SPARC secreted protein acidic and rich in cysteine

The PI3K/AKT/mTOR signaling pathway is crucial to many aspects of cell differentiation, growth, apoptosis, and mobility.^124–126^ Ample evidence has concluded that PI3K/AKT pathway mainly promotes the differentiation of diverse cells into CAFs (Fig. 3). For instance, tumor-derived exosomal miRNA-21, which directly targets PTEN, drove hepatic stellate cell differentiation into CAFs by downregulating PTEN and activating PDK1/AKT signaling pathway.^55^ BMP2 activated the PI3K/AKT and MEK/ERK signaling pathways and induced the transition from pericytes to CAFs, and Noggin (BMP signaling pathway inhibitor) inhibited PI3K/AKT and MAPK signaling pathways and reversed the pericyte–CAFs transition.^127^ The Notch signaling pathway also promoted CAF differentiation from human bone MSCs via AKT pathway.^128^ In addition, there could be a potential correlation between CAF survival and AKT signaling pathway. B7-H3 has been recognized as a co-stimulatory molecule in immune responses.^129^ In renal cell carcinoma, B7-H3 silencing increased apoptosis and prevented the cell cycle process and simultaneously inhibited AKT phosphorylation,^130^ suggesting that AKT pathways might play a role in promoting CAF proliferation and in inhibiting the apoptosis induced by B7-H3. In another study, overexpression of Noggin in CAFs decreased CAF proliferation.^131^ Further, the PI3K/AKT signaling pathway affected the CAF motility. GW4064, as an activator of farnesoid X receptor (FXR), significantly reduced cell migration, and this inhibition was also found in cells expressing wild-type AKT.^132–135^ Interestingly, the PI3K/AKT inhibitor LY294002 significantly potentiated the inhibitory effects mediated by GW4064,^132^ illustrating that PI3K/AKT signaling pathway was involved in the CAF motility mediated by FXR.

#### PI3K/AKT/mTOR signaling pathway-mediated crosstalk of CAFs with cancer cells and its targeted therapy

Many studies on the PI3K/AKT/mTOR pathway in CAFs have shown that activation of this cascade promoted various cancer behaviors, especially cell proliferation (Fig. 3). The fact that PI3K/AKT signaling pathways regulated CAF-mediated cancer cell proliferation in oral,^136^ lung,^137,138^ gastric,^139^ colon,^140^ endometrial,^141^ and anal^142^ cancers. Mechanistically, in gastric cancer, a neutralizing antibody against Nodal attenuated CAF-induced cancer cell proliferation through Nodal-induced activation of the Smad2/3/AKT signal axis.^139^ In another study, blockade of PTEN phosphorylation by siRNA led to the promotion of colon cancer cell proliferation upon stimulation with CXCL12 through the activation of PI3K/AKT signaling pathway.^140^ In contrast, Subramaniam et al. found that a specific PI3K inhibitor (LY294002) reversed the CAF-mediated cell proliferation in endometrial cancer.^141^ These findings suggest that the role of AKT signaling axis in various cancers seems to be tumor/tissue-specific and/or that different inhibitors affect different signal transduction pathways to promote cell proliferation. These observations raise the question: Do different inhibitors attenuate CAF-mediated proliferation via PI3K/AKT signaling pathway in the same type of tumor? In general, blocking vascular cell adhesion molecule-1 (VCAM-1) by siRNA^138^ and inhibiting IL-22 with an anti-IL-22 antibody,^137^ CAF-CM-promoted proliferation was attenuated via factors downstream of PI3K/AKT signaling cascade in the same type of lung cancer.

In addition, in colorectal cancer, CAFs increased the adhesion of cancer cells to endothelial cells and the migration of cancer cells in liver or lung metastasis by upregulating CD44 through HGF/MET/AKT signal pathway.^143^ VM was reported to be facilitated by the cancer cells with sufficient plasticity to form vascular networks for the perfusion of rapidly growing tumors and metastases.^144,145^ Kim et al. provided data showing that CAF-CM-induced VM was closely associated with a high level of erythropoietin-producing human hepatocellular receptor A2 (EphA2).^146^ Interestingly, both an EphA2 inhibitor (siRNA) and a PI3K inhibitor (LY294002) decreased VM induced by CAF-CM and suggested that the EphA2/PI3K or HGF/PI3K signaling pathway was involved in CAF-CM-mediated VM,^146,147^ implying that both EphA2 and HGF might be potential therapeutic targets for cancer anti-vascular treatment in gastric cancer. Of note, downregulation of CAF-derived secreted protein acidic and rich in cysteine (SPARC) can lead to dedifferentiation of gastric cancer cells to CD44^+^/CD24^−^ cancer stem cell (CSC)-like cells, and the AKT/mTOR and MEK/ERK signaling pathways might be involved in these processes,^148^ indicating that CAF-derived SPARC maintained tumor stemness through the AKT/mTOR signaling pathway. Further, both miRNAs and lncRNAs, the two most studied classes of noncoding RNAs (ncRNAs), are crucial regulators of gene expression and interact closely with the PI3K/AKT/mTOR pathway during oncogenesis.^136,149–151^ For instance, in colorectal cancer, CAFs upregulated lncRNA UCA1 in cancer cells and collaborated with mTOR to suppress the miR-143, thereof leading to an increase in KRAS protein and resulting in regulation of the EMT and cell invasion and migration.^152^ Similar to the EMCT discussed above, Ogier et al. provided data showing that 7E3 blocked neuregulin 1 (NRG1)-mediated HER3 and AKT/MAPK signals to inhibit tumor growth in pancreatic cancer,^153^ demonstrating that NRG1 expressed by CAFs and cancer cells is an EMCT candidate. Overall, the CAF-mediated PI3K/AKT signaling pathway regulated cell proliferation, migration, VM, and stemness, and both miRNAs and lncRNAs were involved in this signal cascade. Although various PI3K/AKT inhibitors have been used in many studies, maximizing their utility in CAF-targeted therapy remains challenging. Optimization of the tumor-type selection strategies, the EMCT, and combinatory approaches will help to improve the efficacy of these agents.

### MAPK signaling pathway

#### MAPK signaling pathway in CAFs and its targeted therapy

MAPK signaling pathways comprises signaling cascades involving three major kinases: ERK, c-Jun-N-terminal kinase (JNK), and p38 (MAPK14).^154,155^ Components of the MAPK pathways respond to various input signals, including cytokines, chemokines, growth factors, and stress, etc., signals. Therefore, the MAPK pathway is divided into mitogen- and stress-activated MAPK pathways, with classical representatives being ERK as the mitogen-responsive MAPKs and JNK and p38 as the stress-responsive MAPKs.^156,157^ Once the phosphorylation of ERK1/2, JNK1/2/3, or p38 is induced by an upstream cascade, these kinases are translocated into the nucleus where they activate transcription factors, subsequently leading to the regulation of gene expression.^157,158^

First, it was reported that miR-211 directly targeted the insulin-like growth factor 2 receptor to activate the MAPK signaling pathway, resulting in CAF generation.^40^ Gastric cancer cell-derived exosomes induced pericytes to form CAFs by activating PI3K/AKT and MEK/ERK pathways; however, BMP pathway inhibition reversed the cancer exosome-induced CAF transition.^127^ Surprisingly, CAFs could utilize lipids endogenously synthesized by a gold nanoparticle to induce the expression of lipogenesis genes such as fatty acid synthase (FASN), sterol response element-binding protein 2, and fatty acid-binding protein 3, and thus maintain a quiescent phenotype.^159^ In addition, Ando et al. showed that eicosapentaenoic acid, a polyunsaturated fatty acid, decreased the expression of IL-6 and VEGF in CAFs by inhibiting the ERK pathway, thereby reducing the cancer angiogenesis in vitro.^160–162^ Indeed, fatty acids are necessary for the basic functions of nearly all cell types including CAFs,^163^ and FASN is a key lipogenic enzyme in the biogenesis of fatty acids that generates palmitate from malonyl-CoA and acetyl-CoA in the presence of nicotinamide adenine dinucleotide phosphate.^164,165^ Intriguingly, 17β-estradiol (E_2_) and G1 upregulated FASN involved in the metabolism of fatty acids in CAFs via EGFR/ERK signaling cascade.^166^ In fact, MAPK signal was found to be involved not only in the metabolism of fatty acids but also in glycolysis in CAFs. We have found that CAF-lncRNA H19 regulated the levels of phosphorylated ERK, JNK, and p38 and further promoted glycolysis reprogramming in OSCC,^52^ demonstrating that the activated MAPK signaling may contribute to glucose metabolism in CAFs. In human lung cancer, CAFs displayed significantly higher migration activity in response to PDGF-BB than fibroblasts derived from noncancerous tissues and were presumed to be more dependent on ERK1/2 signaling for enhanced migration activity.^167^ Similarly, in another study, Eck et al. found that compared to the NFs in the mammary tissue, CAFs expressed increased CXCR4 and that AMD3100 (a CXCR4 inhibitor) suppressed the phosphorylation of ERK1/2 caused by CXCL12, subsequently leading to less invasive and migratory CAF phenotypes.^168^ In addition, it was reported that tissue inhibitor of metalloproteinase-1 (TIMP-1) could enhance prostate CAFs proliferation and migration in vitro and activate the ERK1/2 signaling pathway in CAFs.^169^ However, TIMP-1 significantly promoted CAF proliferation and motility but not the proliferation of tumor cells in prostate cancer,^169^ suggesting that the TIMP-1-mediated accumulation of prostate CAFs likely resulted from both enhanced infiltration and expansion of prostate CAFs within the tumors. With regard to ECM, upregulated Snail1 was found in CAFs, and it was required for the fibrogenic response of CAFs exposed to a stiff matrix.^170^ Mechanistically, increased ERK2 activity augmented the nuclear accumulation of Snail1 to decrease cytosolic proteasome degradation, and Snail1 affected the expression and activity of YAP1 in CAFs exposed to a stiff matrix.^170^

#### MAPK signaling pathway-mediated crosstalk of CAFs with cancer cells and its targeted therapy

MAPK signaling pathways, as ubiquitous signal transduction, regulate almost all aspects of cellular function in cancers.^157,171^ For cell proliferation mediated by CAFs in MAPK signal, endometrial cancer cell proliferation was prompted significantly by CAF-CM compared to NF-CM through the phosphorylated ERK, and it could be reversed by U0126 (an ERK selective inhibitor).^141^ Similarly, CAF-derived epiregulin significantly enhanced cancer cell proliferation through a downstream effector of ERK, and ERK inhibitors U0126 and PD98059 counteracted epiregulin-induced promotion of tumor growth in colitis-associated cancer.^172^ These findings suggest that MAPK/ERK signaling pathway is evolutionarily conserved and that U0126 is a highly effective depressant of this cascade. Notably, it was shown that Twist1 exhibited a dual role in CAFs and cancer cells in the EMT process: on the one hand, Twist1 promoted the expression and secretion of CXCL12 from CAFs, and its knockdown in CAFs inhibited tumor growth; on the other hand, activated CXCL12/CXCR4 signaling promoted EMT process through ERK/AKT-Twist1-MMP1/E-cadherin pathway in esophageal cancer cells.^173^

**Fig. 4:**
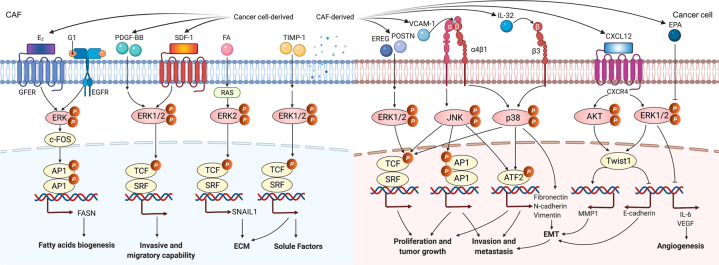
MAPK signaling pathway in CAFs and the crosstalk of CAFs with cancer cells. In CAFs, by E_2_ and G1, the EGFR/ERK signaling upregulated FASN expression for the metabolism of fatty acids. The PDGF-BB and SDF-1 could stimulate the higher invasive and migratory capability of CAFs via ERK1/2 phosphorylation. FA activated RAS upregulating SNAIL1 via ERK2 signaling, which mediated the fibrogenic response of CAFs. TIMP-1 enhanced CAF proliferation and migration and activated ERK1/2 signaling pathway in CAFs by the production of soluble factors. In the crosstalk between CAFs and cancer cells, CAF-derived EREG and POSTN could enhance the cancer cell proliferation and tumor growth by the downstream effector of ERK1/2. VCAM-1 and CAF-derived IL-32 increased the proliferation, invasion, metastasis, and EMT in cancer cells by activating p38/MAPK signaling pathway. Activated CXCL12/CXCR4 signal promoted EMT process through ERK/AKT-Twist1-MMP1 pathway. EPA decreased the expression of IL‑6 and VEGF secretion in CAFs by inhibition of ERK phosphorylation, thereof affecting angiogenesis. E_2_ 17β-estradiol, G1 1-(4-(6-bromobenzo[1,3]dioxol-5-yl)-3*a*,4,5,9*b*-tetrahydro-3*H*-cyclopenta[*c*]quinolin-8-yl)-ethanone, 1-[(3a*S*,4*R*,9*bR*-rel)-4-(6-bromo-1,3-benzodioxol-5-yl)-3*a*,4,5,9*b*-tetrahydro-3*H*-cyclopenta[*c*]quinolin-8-yl]-ethanone, PDGF-BB platelet-derived growth factor-BB, FA focal adhesions, TIMP-1 tissue inhibitor of metalloproteinase-1, AP1 activating protein 1, FASN fatty acid synthase, SRF serum response factor, TCF ternary complex factor, POSTN periostin, EPA eicosapentaenoic acid, ECM extracellular matrix, EMT epithelial–mesenchymal transition

Of note, one of the most important characteristics of the MAPK pathway in the crosstalk between CAFs and cancer cells (Fig. 4), we propose, is the extensive cross-signaling between MAPK pathways and other cascades, such as PI3K/AKT signal, JAK/STAT cascade, and TGF-β pathway, in various cancers.^127,147,174^ For instance, blocking VCAM-1 suppressed proliferation and invasion of CAF-CM-treated cancer cells by activating the MAPK/AKT signaling pathway.^138^ Consistently, CAFs secreted urokinase plasminogen activator (uPA) to promote cancer cell proliferation, migration, and invasion through PI3K/AKT and MAPK/ERK signaling pathways in esophageal squamous cell carcinoma (ESCC).^175^ In another study, CAFs promoted the viability of neuroblastoma cells by increasing their proliferation and inhibiting their apoptosis through co-activation of the JAK2/STAT3 and MEK/ERK1/2 signaling pathways.^176^ In a mouse model of neuroblastoma, inhibition of JAK2/STAT3 and MEK/ERK/1/2 by ruxolitinib and trametinib treatment, respectively, potentiated the tumor response to etoposide and suppressed tumor progression.^176^ In summary, MAPK signaling pathways have great potential as targets in cancer therapy, and currently, the most extensively studied MAPK signal is the ERK pathway. An alternative approach, unlike the EMCT, we suspect, which is supported by the observation with CAF-derived epiregulin,^172^ CAF-derived periostin^177^ or CAF-secreted uPA,^175^ is that the dual targeting of the key biomarker in CAFs and its vital downstream effector of MAPK signaling axis in cancer cells may optimize the efficacy of blocking the crosstalk between CAFs and cancer cells in targeted therapy.

In addition, MAPK/p38 or MAPK/JNK signaling pathway also plays important roles in the crosstalk of CAFs with cancer cells (Fig. 4). Blockage of MAPK/p38 pathway diminished IL-32-induced EMT markers, cell invasion, and metastasis in breast cancer.^178^ Li et al. found that DSF/Cu increased cellular ROS levels and activated the apoptosis-related MAPK pathway without inducing a significant change in JNK or p38 expression.^179^ However, stress-activated MAPK pathways, including JNK cascade and p38 pathway, continued to exert the complementary functions in CAF-targeted MAPK signaling pathways in cancer treatment.

### Wnt signaling pathway

#### Wnt signaling pathway in CAFs and its targeted therapy

Wnt signaling pathway includes 19 Wnt ligands and more than 15 receptors, which can be classified into canonical and noncanonical signaling pathways.^116,180^ In the canonical cascade, in the absence of Wnt ligands, cytoplasmic β-catenin combines with Axin complex and phosphorylates by glycogen synthase kinase 3β (GSK3β), leading to β-catenin degradation in the cytoplasm via β-TrCP200 ubiquitination.^181,182^ Conversely, in the presence of Wnt ligands, including Wnt1, Wnt2, and Wnt3a, the ligands combine with Fzd/LRP (LDL-receptor-related protein) receptors, and then, LRP receptors are phosphorylated by GSK3β, thereby causing the release of β-catenin from the Axin complex and translocation from the cytoplasm into the nucleus for targeted gene expression, including CD44, c-Myc, and cyclin D1.^183^ β-Catenin is not involved in the noncanonical Wnt signaling process. Through the binding of FZD receptors or ROR1/ROR2/RYK coreceptors, Wnt/RCP and Wnt/Ca^2+^ signaling cascades are activated for transcriptional responses and/or cytoskeletal rearrangement.^184,185^

In Wnt/β-catenin signaling, CAF-derived β-catenin became a major concern, as it is seemed to be a relatively early-stage event in carcinogenesis. For instance, many CAFs infiltrated into and/or around invasive tissue in the presence of high β-catenin levels in human melanoma.^186^ Through a new conditional gene knockout system (Col1α2-CreER mouse), β-catenin was depleted in dermal fibroblasts, causing cell cycle arrest and suppressing cell proliferation and chemical factor and ECM protein production.^186^ Similarly, in colorectal cancer, Mosa et al. generated a Wnt3^HA/HA^APC^min/+^ mouse model and demonstrated a direct role of Wnt signaling in fibroblast activation, contractility, and CAF phenotypic plasticity.^187^ Importantly, β-catenin ablation reduced the expression of PDGFRα and FSP1 with no obvious cytoskeletal rearrangement in stromal fibroblasts,^188^ noting that other signaling pathways might be implicated in the process of cytoskeletal rearrangement mediated by noncanonical Wnt signaling cascade. It has been shown that β-catenin also forms a β-catenin/E-cadherin complex that contributes to the motility and migration of fibroblasts.^189^ In HNSCC, periostin is highly produced and secreted by CAFs.^190^ CAF-derived periostin was found to be a potential ligand for protein tyrosine kinase 7 (PTK7) and was correlated with Wnt/β-catenin signal activation.^190^ In addition, unlike DKK1/2/4, which suppressed the Wnt/β-catenin signaling cascade, DKK3 neither interacted with LRP5/6 nor fulfilled the antagonistic role of a bona fide member of the DKK family in the canonical Wnt signaling pathway.^191,192^ However, DKK3 decreased the stability of Kremen to increase LRP6 membrane localization and stabilization of β-catenin.^191,193^ Interestingly, in CAFs, heat-shock factor-1 interacted with the DKK3 locus and upregulated the expression of DKK3,^194^ indicating that DKK3 might be a target for blocking of the Wnt/β-catenin signaling.

#### Wnt signaling pathway-mediated crosstalk of CAFs with cancer cells and its targeted therapy

Wnt signaling pathway is aberrantly activated in various cancers, including melanoma,^186^ esophageal,^195^ head and neck,^190,196^ breast,^193^ gastric,^197^ liver,^198^ ovarian,^193^ and colorectal cancers,^193,199^ and its genetic alterations are frequent, at ~66.55%, in cancers.^200^ What distinguishes the Wnt signaling pathway in CAFs from other pathways? Notably, in contrast to the studies on mutations in APC, RNF43, ZNRF3, AXIN1/2, and CTNNB1 detected in human colorectal adenocarcinoma,^201^ endometrial cancer,^202^ HCC,^203^ and gastric cancer,^204^ few studies have been published related to their alterations of these genes in CAFs. To further address the role of cancer cell mutations in CAFs, using a 3D coculture model, Zhou et al. found that melanoma growth was suppressed by CAF deactivation induced by β-catenin ablation, which led to the reduced production of paracrine factors and ECM proteins.^188^ Similarly, CAF-derived periostin promoted the CSC phenotype, tumor progression, and metastasis via canonical Wnt/β-catenin signaling pathway in HNSCC.^190^ Mechanistically, CAF-derived periostin bound to PTK7 on the cancer cell membrane and transferred the signals to disheveled proteins by LRP6, thereby inducing the phosphorylation of GSK3β and the hypophosphorylation of β-catenin, leading to the translocation of β-catenin from the cytoplasm to the nuclei.^190^ However, it was reported that β-catenin-mediated Wnt signaling was dispensable for the function of CAFs in ECM remodeling and promoting cell proliferation and invasion in breast cancer.^193^ This suggests that Wnt/β-catenin signaling pathway affects the crosstalk of CAFs and cancer cells in highly specific tumor types. Interestingly, by generating Wnt-independent tumor organoids, which secreted the Wnt antagonist Sfrp1, Mosa et al. found that Sfrp1 or genetic depletion of β-catenin strongly decreased the number of cancer-associated myofibroblasts (myCAFs: α-SMA^+^/Acta2^+^).^187^ Coculture of this tumor organoid with inflammatory CAFs (iCAFs: IL-6^+^/Tnfa^+^/IL-1a^+^) resulted in the upregulation of Vim and Zeb1, while myCAFs reverse this upregulation,^187^ indicating that the EMT process could be induced by Sfrp1 and that tumor behaviors were differentially regulated via Wnt signaling pathway in specific CAF subtypes.

**Fig. 5:**
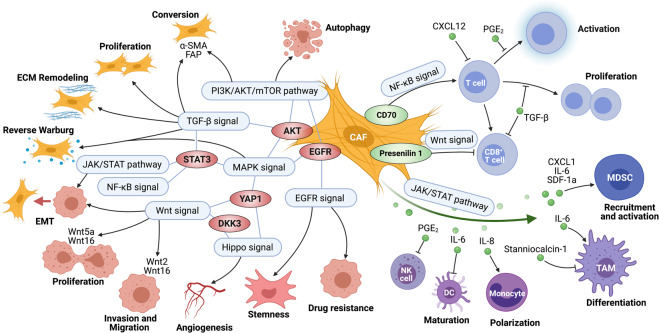
Crosstalk of different signaling pathways among CAFs, cancer cells, and immune cells. A reservoir of biological behaviors of CAFs, including CAFs generation, proliferation, ECM remodeling, and energy metabolism, etc. were regulated by several major signals like TGF-β and PI3K/AKT/mTOR signaling pathways. Importantly, CAF-mediated signaling pathways like JAK/STAT, Wnt, Hippo, MAPK, EGFR, and NF-κB signal were widely involved in cancer cells proliferation, stemness, invasion, migration, metastasis, angiogenesis, epithelial–mesenchymal transition (EMT) process, and therapeutic resistance. CAF-mediated signaling pathways did not always display with individual effects, but commonly crossed to each other to form a signaling network in cancer progression by the cross-connections such as STAT3, AKT, and YAP1. As the great source of cytokines, chemokines, and growth factors, CAF-secreted factors, including TGF-β1, IL-6, IL-8, CXCL1, CXCL12, and PGE_2_, etc., affect proliferation and activation of T cell, recruitment and activation of myeloid-derived suppressor cells (MDSCs), differentiation, and polarization of monocytes/macrophages, etc. PGE_2_ prostaglandin E_2_

In addition, CAF-derived Wnts can lead to cell growth and other biological functions of cancer cells (Fig. 5). For example, Wnt2 protein secreted by CAFs enhanced cell invasion and migration in colorectal cancer^205^ and angiogenesis by shifting the balance towards proangiogenic signaling in colon cancer.^206^ It is likely that treatment with CAF-CM and an elevated autophagy rate augmented the levels of β-catenin and P-GSK3β, which are the key proteins in the Wnt/β-catenin pathway, thereby promoting tumor progression.^207^ Notably, the upregulation of Wnt proteins in CAFs was explainable with both intrinsic and extrinsic aspects. On the one hand, Wnt5a was enriched by the loss of H3K27me3 in CAFs, and inhibition of secreted Wnt5a from CAFs suppressed cancer cell growth and migration in gastric cancer.^208^ On the other hand, Taxotere treatment enhanced Wnt16 expression in CAFs and this in turn might have contributed to the proliferation, invasion, and chemoresistance of breast cancer cells.^209^ Taken together, these findings show that the attenuated Wnt signaling cascade in CAFs could contribute as a suppressor of tumor progression, in a manner similar to that described for reduction in tumor cell-intrinsic Wnt signaling activity; however, more studies are required to dissect the underlying potential mechanisms and genetic factors related to CAF-mediated Wnt signaling in cancer progression.

### JAK/STAT signaling pathway

#### JAK/STAT signaling pathway in CAFs and its targeted therapy

JAK/STAT signaling pathway is a signal cascade stimulated by many kinds of cytokines and consists of a host of ligands and several tyrosine kinase-related receptors with four tyrosine kinase JAK and seven transcription factor STAT family members, suppressors of cytokine signaling proteins, and multiple STAT-dependent operons.^210,211^ JAK enzymes share a common domain structure consisting of seven JAK-homology domains.^212^ Typically, cytokines, chemokines, and/or growth factors integrate with tyrosine kinase-related receptors, and the latter recruits JAK, activating receptor and JAK. The phosphorylated tyrosine on the receptor molecule binds with the SH2 site of STAT.^213^ STAT binding to the receptor triggers the tyrosine phosphorylation of STAT, leading to STAT dimer formation and its translocation into the nuclei where it targets gene expression.^214^

JAK/STAT signaling pathway is constitutively activated in CAFs. In TME, CAF-derived cytokines, including IL-6, IL-10, IL-11, and IL-22, act as ligands for JAK/STAT signal cascade (Table 3). Intriguingly, IL-6, as a pro-inflammatory cytokine, partnered with GP130 to activate STAT3, while IL-10, as an anti-inflammatory cytokine, did not interact GP130 but promoted the phosphorylation of STAT3,^215–217^ demonstrating that different cytokines are likely to activate the same STAT protein. Actomyosin contractility plays a key role in ECM remodeling by CAFs to permit cell migration. GP130-IL6ST signaling influenced JAK1-derived actomyosin-mediated contractility through the phosphorylation of MLC2 in CAFs and promoted ECM remodeling.^218,219^ Consistently, cytokine oncostatin M not only promoted actomyosin-mediated contractility and ECM remodeling by CAFs through GP130-IL6ST, JAK1, and ROCK signal axes but also induced CAF generation through the JAK/STAT signaling pathway.^220^ In addition, aberrant DNA methylation contributed to the maintenance of the phenotype of CAFs via the JAK/STAT cascade.^221^ Since STAT3 acetylation caused the epigenetic modification-dependent loss of SRC homology phosphatase-1 (SHP-1) and dephosphorylates JAK1, SHP-1 knockout led to the sustained constitutive phosphorylation of JAK1 and STAT3, which maintained the contractility- and invasion-promoting properties of CAFs.^221,222^ To attenuate the effect of specific cytokines on JAK/STAT signal, therapeutic approaches, including blocking cytokine antibodies or inhibitors, are warranted to identify the tumor-promoting roles of CAFs. Targeted inhibition, such as that induced by 5-azacytidine and ruxolitinib treatment, resulted in the sustained abrogation of JAK1/STAT3 phosphorylation and rescued SHP-1 expression, thereby inhibiting the tumor-promoting invasive phenotypes of CAFs.^223,224^ In an analysis of miRNAs in CAFs through JAK/STAT signaling pathway, miR-210 increased the expression of matrix metalloproteinase 9 (MMP9), FGF2, and VEGFA by activating the JAK2/STAT3 signaling pathway for proangiogenesis and ten-eleven translocation 2 was identified as the target of miR-210 in CAFs, which was implicated in proangiogenic switching.^225^ In addition, p53 was reported to regulate the CAF properties through STAT3 signaling, and CAF activation, migration, and invasion could be clearly inhibited by Stattic (Y705), an inhibitor of STAT3.^226^

**Table 3:** CAF-secreted factors and their roles in cancers

Factors	Cancer type	Recipient cell	Biological function	Activated pathway	Refs.
Cytokine					
IL-1β	OSCC	Cancer cell	Promotes cell growth	NF-κB pathway	^445^
IL-1β	OSCC, BC	Cancer cell	Promotes cell invasion	IL-1β/ IL-1R pathway	^446,447^
IL-6	ESCA	Cancer cell	Promotes cell chemoresistance	STAT3/NF-κB pathway	^218,448^
IL-6	LC, HCC, PRAD	Cancer cell	Promotes cell metastasis and chemoresistance	JAK2/STAT3 pathway	^219,449–451^
IL-6	GC, BLCA, GBC, BC, UCEC	Cancer cell	Promotes cancer progression	JAK/STAT3 pathway	^230,448,452–456^
IL-6	HNSC	Cancer cell	Promotes cancer progression	Integrin αvβ3/NF-κB pathway	^295^
IL-6	CRC	Monocyte	Promotes cell adhesion	ERK1/2 pathway	^341^
IL-6	HCC	Cancer cell	Promotes stem cell-like properties	STAT3/Notch pathway	^457^
IL-6	HCC	Neutrophils	Regulates cell survival, activation, and function	STAT3/PD-L1 pathway	^458^
IL-8	CRC	Monocyte	Recruits monocyte and promotes its polarization	IL-8/CXCR2 pathway	^341^
IL-8	GC	Cancer cell	Promotes cisplatin resistance	NF-κB pathway	^459^
IL-11	GC, LC	Cancer cell	Promotes cell chemoresistance and metastasis	JAK/STAT3 pathway	^174,460,461^
IL-17a	GC	Cancer cell	Promotes cell migration and invasion	JAK2/STAT3 pathway	^232^
IL-22	GC	Cancer cell	Promotes cell invasion	STAT3 and ERK pathway	^462^
IL-23	BC	Cancer cell	Promotes cell invasion and metastasis	p38/MAPK pathway	^178^
IL-25	BC	Cancer cell	Suppresses cell metastasis	ND	^463^
IL-33	HNSC	Cancer cell	Promotes cell migration and invasion	ND	^464^
Chemokine					
CXCL5	CRC	Cancer cell	Promotes tumor immunosuppression	PI3K/AKT pathway	^465^
CXCL9	OSCC	Cancer cell	Suppresses cell apoptosis	CXCL9/CXCR3 pathway	^466^
CXCL12	CRC	Cancer cell	Promotes cell metastasis	PI3K/AKT pathway	^140^
CXCL12	BC	Cancer cell	Promotes cell invasion	TGF-β pathway	^305^
CXCL12	OC	Cancer cell	Promotes EMT and cisplatin resistance	Wnt/β-catenin pathway	^467^
CXCL12	LC, HNSC, PAAD, GC	Cancer cell	Promotes cancer progression	CXCL12/CXCR4 pathway	^468–472^
CCL3	PRAD	Cancer cell	Promotes cell migration and invasion	JAK/STAT3 pathway	^473^
CCL5	GC	Cancer cell	Promotes cell progression	CCL5/CCR5 pathway	^474^
CXCL2	LUAD	Cancer cell	Promotes cancer immunosuppression	ND	^475^
CXCL16	BC	Monocyte	Promotes the recruitment of monocyte	ND	^476^
SDF-1	PAAD, CRC	Cancer cell	Promotes cell gemcitabine resistance and metastasis	SDF-1/CXCR4 pathway	^477,478^
SDF-1	UCEC	Cancer cell	Promotes cancer progression	PI3K/AKT and MAPK/ERK pathway	^479^
Growth factors					
	CRC	Cancer cell	Promotes cell cetuximab resistance	MAPK pathway	^253^
FGF1	OC	Cancer cell	Promotes cancer progression	FGF/FGFR pathway	^480^
FGF2	BC, LC, CRC	Cancer cell	Promotes cancer progression	FGF/FGFR pathway	^481–483^
FGF9	GC	Cancer cell	Promotes cell invasion	ERK and AKT pathway	^484^
GPER	BC	Cancer cell	Promotes cell proliferation	GPER/EGFR/ERK pathway	^248^
GDF15	PRAD	Cancer cell	Promotes cancer progression	TGF-β/GDF15 pathway	^485^
HGF	CRC, GC, HNSC	Cancer cell	Promotes cell progression and metastasis	HGF/c-Met pathway	^143,452,486^
HGF	GC	HUVEC	Promotes angiogenesis	PI3K/AKT and ERK1/2 pathway	^147^
HGF	HCC	Cancer cell	Promotes cell chemoresistance	MEK-ERK1/2 pathway	^487^
HGF	HCC	Cancer cell	Promotes cell plasticity	c-Met/FRA1/HEY1 pathway	^488^
HGF	LC	Cancer cell	Promotes cell chemoresistance	HGF/IGF-1/ANXA2 pathway	^489^
IGF-1					
IGF-1	BLCA	Cancer cell	Promotes cell chemoresistance	IGF-1/AKT pathway	^355^
IGF-1	CRC	Cancer cell	Promotes cell survival	IGF-1/IGF1R pathway	^490^
IGF-2	AC	Cancer cell	Promotes cell growth	PI3K/AKT/mTOR pathway	^142^
IGF-2	LC	Cancer cell	Promotes cell chemoresistance	IGF-2/IGF‐1R pathway	^491^
TGF-β1	BC, PRAD	Cancer cell	Promotes cell proliferation and migration	TGF-β/Smad pathway	^492,493^
VCAM-1	LC	Cancer cell	Promotes cell growth and invasion	AKT and MAPK pathway	^138^
VCAM-1	GC	Cancer cell	Promotes cell invasion	JAK/STAT1 pathway	^494^
Others					
ADAM17	BC	Cancer cell	Promotes cell proliferation	EGFR, AKT, and ERK pathway	^495^
Activin A	CRC	Cancer cell	Promotes cell migration and invasion	ND	^496^
ANXA3	LC	Cancer cell	Promotes cell cisplatin resistance	ANXA3/JNK pathway	^497^
Asporin	GC	CAF	Promotes CAF migration	ND	^498^
CD9, GAS6	GC	Cancer cell	Promotes cell migration	ND	^499,500^
CDH-11	BC	Cancer cell	Promotes cell migration	ND	^501^
CLEC3B	CRC	Cancer cell	Promotes cell migration	ND	^502^
Collagen	BC	Cancer cell	Promotes cell chemoresistance	PI3K/AKT pathway	^503^
Collagen	PDAC	Cancer cell	Promotes cell growth and migration	Integrin β1/FAK pathway	^504^
Fatty acids	CRC	Cancer cell	Promotes cell migration	ND	^505^
FN	PRAD	Cancer cell	Promotes cell migration	ND	^313^
Gal1	GC	Cancer cell	Promotes cancer progression and metastasis	ND	^506^
Galectin-1	GC	HUVEC	Promotes angiogenesis	ND	^507^
Grem1	BC	Cancer cell	Promotes cancer progression	BMP/Smad pathway	^508^
HIAR	BC	Endothelial cell	Promotes angiogenesis and cell migration	VEGF/VEGFR pathway	^509^
HIC-5	ESCA	Cancer cell	Promotes cell metastasis	ND	^510^
HMGB1	BC	Cancer cell	Promotes cell stemness and tumourigenicity	HMGB1/TLR4 pathway	^511^
Lactate	BC	Cancer cell	Promotes cell invasion	TGF-β1/p38/MAPK pathway	^512^
LOX	GC	Cancer cell	Promotes cell growth	ND	^513^
LOXL2	CRC	Cancer cell	Promotes cell invasion and metastasis	FAK pathway	^514^
Lumican	GC	Cancer cell	Promotes cell tumorigenesis and metastasis	Integrin β1/FAK pathway	^515^
M-CSF	PDAC	Monocyte	Promotes TAM phenotype	ND	^516^
MFAP5	OSCC	Cancer cell	Promotes cell proliferation and migration	MAPK and AKT pathway	^517^
MK	OSCC	Cancer cell	Promotes cell cisplatin resistance	ND	^518^
MMP2	CESC, OSCC	ECM	Promotes cancer invasion	ND	^519,520^
NAB2	HNSC	Cancer cell	Promotes cell growth	ND	^521^
Notch3	HCC	Cancer cell	Promotes cell self-renewal	ND	^522^
PAI-1	ESCA	Cancer cell	Promotes cell cisplatin resistance	AKT and ERK1/2 pathway	^523^
POSTN	GC	Cancer cell	Promotes cell growth	ERK pathway	^177^
POSTN	HNSC	Cancer cell	Promotes cell stemness	PTK7-Wnt/β-catenin pathway	^190^
POSTN	CESC	Endothelial cell	Promotes cancer metastasis	FAK/SRC pathway	^524^
POSTN	OC, OAC	Cancer cell	Promotes cell cisplatin resistance and invasion	PI3K/AKT pathway	^525,526^
Pyruvate	Lymphoma	Cancer cell	Promotes cell survival	ND	^361^
RANKL	OSCC	Osteoclast	Promotes bone invasion	ND	^527^
SNAI1	LC	Cancer cell	Promotes cell migration and invasion	ND	^528^
TNFSF4	LUAD	Cancer cell	Promotes chemoresistance	NF-κB/BCL-XL pathway	^529^
uPA	ESCA	Cancer cell	Promotes cancer progression	PI3K/AKT and ERK pathway	^175^
WNT2	CRC	Cancer cell	Promotes cell invasion and migration	ND	^205,206^

*AC* anal cancer, *ANXA3* annexin A3, *BC* breast cancer, *BLCA* bladder cancer, *CAF* cancer-associated fibroblast, *CDH-11* cadherin-11, *CESC* cervical and endocervical cancer, *CLEC3B* c-type lectin domain family 3 member B, *CRC* colon adenocarcinoma, *ECM* extracellular matrix, *EGF* epidermal growth factor, *EGFR* epidermal growth factor receptor, *ERK* extracellular regulated protein kinases, *ESCA* esophageal carcinoma, *FGF* fibroblast growth factor, *FN* fibronectin, *Gal1* galectin-1, *GAS6* growth arrest specific protein 6, *GBC* gallbladder cancer, *GC* gastric cancer, *GDF15* growth differentiation factor 15, *GPER* estrogen receptor, *Grem1* gremlin 1, *HCC* hepatocellular carcinoma, *HGF* sepatocyte growth factor, *HIAR* hypoxia-induced angiogenesis regulator, *HIC-5* hydrogen peroxide-inducible clone 5, *HMGB1* high-mobility group box 1, *HNSCC* head and neck squamous cell carcinoma, *HUVEC* human umbilical vein endothelial cell, *IGF* insulin-like growth factor, *IL* interleukin, *JAK* janus tyrosine kinase, *LC* lung cancer,LOX lysyl oxidase, *LOXL2* lysyl oxidase-like 2, *LUAD* lung adenocarcinoma, *MAPK* mitogen-activated protein kinase, *MEK* mitogen-activated protein kinase, *MFAP5* microfibril associated protein 5, *MK* midkine, mTOR mammalian target of rapamycin, *ND* not determined, *NF-κB* nuclear factor kappa-B, *OAC* esophageal adenocarcinoma, *OC* oarian cancer, *OSCC* oral squamous cell carcinoma, *PAAD* pancreatic adenocarcinoma, *PDAC* pancreatic ductal adenocarcinoma, *PAI-1* plasminogen activator inhibitor 1, *PD-L1* programmed cell death 1 ligand 1, *PI3K* PI3 kinase, *POSTN* periostin, *PRAD* prostate adenocarcinoma, *PTK* tyrosine protein kinase, *RANKL* receptor activator of nuclear factor kappa-B ligand, *SDF-1* stromal cell-derived factor-1, *SPARC* secreted protein acidic and rich in cysteine, *STAT3* signal transducer and activator of transcription 3, *TGF* transforming growth factor, *TNFSF4* tumor necrosis factor ligand superfamily member 4, *UCEC* uterine corpus endometrial carcinoma, *VCAM-1* vascular cell adhesion molecule-1

#### JAK/STAT signaling pathway-mediated crosstalk of CAFs with cancer cells and its targeted therapy

Constitutive activation of JAKs and STATs was first identified as being associated with malignancy,^227^ and accumulating evidence has shown that CAF-mediated JAK/STAT signaling pathway is widely involved in human cancers, including prostate,^228^ lung,^229^ breast,^230^ colorectal,^231^ gastric,^232^ endometrial,^233^ liver,^234^ and oral^235^ cancers, through various tumor biological processes, including in increased cell plasticity, proliferation, migration, invasion, EMT, angiogenesis, and metastasis. Notably, IL-6 represents the most investigated cytokine regulating the crosstalk between CAFs and cancer cells (Table 3). In HCC, CAF-derived IL-6 facilitated HCC cell EMT, which in turn activated the IL-6/IL-6R/STAT3 axis in a positive-feedback loop to promote the expression of TG2 for the acquisition of EMT phenotypes.^234^ IL-6 binding with GP130 could trigger STAT3 activation, and this response could be suppressed by the inhibition of netrin-1.^236^ Netrin-1, as a multifunctional secreted glycoprotein, is highly expressed in colon CAFs, and its blocking antibody (Net1-mAb) inhibited cancer stemness, plasticity, and intercellular signaling between CAFs and cancer cells by suppressing the IL-6/JAK2/STAT3 signaling pathway.^236^

As the upstream of IL-6, epiregulin-induced CAF activation promote EMT by activating IL-6/JAK2/STAT3 signaling axis, which could be inhibited by a JAK2 inhibitor (AG490).^235^ Intriguingly, the migration of melanoma cells was dependent on GP130-IL6ST/JAK1-ROCK signaling; however, although it was not necessary in cancer cells, this signaling pathway was required for CAF-induced ECM remodeling to promote the invasion of squamous cell carcinoma (SCC),^237^ indicating that the targeted therapy of JAK/STAT signaling pathway for SCC invasion might not be the epithelium but CAFs. Further, cytokine signaling of GP130-IL6ST/JAK1 cascade mediated actomyosin contractility in cancer cells and CAFs to promote SCC invasion.^237^ Estrogen in CAF-CM promoted gastric cancer cell proliferation and invasion via IL-6/STAT3 signal axis, and these two processes could be inhibited by an IL-6-neutralizing antibody and STAT3 siRNA, respectively.^236^

In addition, CAF-derived IL-17a significantly promoted the migration and invasion of gastric cancer cells by activating the JAK2/STAT3 signaling pathway, and the effects of CAF-mediated cancer progression were inhibited significantly by treatment with an IL-17a-neutralizing antibody or JAK2 inhibitor (AG490).^232^ In addition, Heichler et al. found that IL-11 was frequently overexpressed in colorectal cancer and acted as a signal transducer and activator of STAT3, which was inversely correlated with patient prognosis.^231^ Taken together, therapeutic agents targeting the JAK/STAT signaling pathway, including blocking antibodies against Netrin-1, cytokines including IL-6, IL-11, and IL-17a, or inhibitors of JAK kinase such as AG490 or STAT activity such as STAT3 siRNA, could be useful agents in antitumor treatment.

### EGFR signaling pathway

#### EGFR signaling pathway in CAFs and its targeted therapy

EGFR belongs to the ErbB family of receptors, which includes ErbB1/EGFR/HER1, ErbB2/HER2/Neu, ErbB3/HER3, and ErbB4/HER4.^238^ ErbB family members can be activated by the following ligands: amphiregulin, betacellulin, EGF, heparin-binding EGF-like growth factor, TGF-α, epiregulin, epigen, and NRGs.^239,240^ Structurally, EGFR family members share a common domain arrangement that comprises a cysteine-rich extracellular domain, a hydrophobic transmembrane domain, and an intracellular tyrosine kinase domain. Specifically, the extracellular region of EGFR is subdivided into four domains, and the intracellular tyrosine kinase domain is highly conserved with variable phosphorylation sites.^241,242^ The EGFR signaling pathway is activated by ligand-induced receptor dimerization, in which the tyrosine residues in the intrinsic kinase domain of one receptor cross-phosphorylate specific residues in the C-terminal tail of the partnering receptor to recruit functional proteins.^243,244^

EGFR is expressed in almost all nonneoplastic cell types in TME, including CAFs, except mature cells in the lymphohematopoietic system.^245,246^ GPER was first reported as a *GPCR* gene in breast cancer,^247^ and Luo et al. found that GPER expression was abundant in breast CAFs.^248^ G15 (a selective GPER antagonist), AG (an inhibitor of EGFR), and U0126 (an inhibitor of ERK1/2) significantly inhibited GPER-mediated proliferation and cell cycle changes in breast CAFs induced by E_2_, G1, and tamoxifen,^248^ implying that GPER/EGFR/ERK signaling pathway was activated in this process. Of note, zinc chloride (ZnCl_2_) increased GPER-targeted CTGF in breast CAFs.^249^ Since CTGF was reported to have a role in the migration of different cell types,^250^ Pisano et al. found that ZnCl_2_-stimulated migration of CAFs was abolished by knockdown of GPER or CTGF, and this migratory response could be rescued by the addition of CTGF.^249^ In mammals, α1,6-fucosyltransferase (FUT8), as the only enzyme that catalyzes core α1,6-fucosylation (CF), was reported to be overexpressed in CAFs in 82% of lung adenocarcinoma cases, and upregulated FUT8 could prompt the CF modification at high levels.^251^ Downregulation of either EGFR or FUT8 reduced the phosphorylation of EGFR; however, the blockade of EGFR signaling was rescued by an EGFR activator in FUT8 down-regulated CAFs,^251^ demonstrating that EGFR activity in CAFs was regulated by this FUT8/CF treatment.

#### EGFR signaling pathway-mediated crosstalk of CAFs with cancer cells and its targeted therapy

Currently, EGFR tyrosine kinase inhibitors (EGFR-TKIs) are being effectively used for anticancer therapy, while CAF-derived survival signaling to cancer cells can modify EGFR-TKI efficacy.^252–256^ In HNSCC, it was reported that EGF-containing fibulin-like ECM protein-1 (FBLN3) was secreted by CAFs but not NFs, and CAF-derived FBLN3 could increase anchorage-independent growth and tumor sphere formation and maintain cancer stemness.^257^ Interestingly, targeting the EGFR signaling pathway with gefitinib effectively inhibited CAF-mediated cancer stemness,^257^ demonstrating that CAF-derived factors such as FBLN3 in the EGFR signal cascade promoted cancer stemness properties are potential therapeutic targets to effectively block CAF-promoted CSC niche formation. In another study, it was shown that FUT8/CF in CAFs prompted the proliferation of cancer cells through EGFR signal cascade in non-small-cell lung cancer,^251^ suggesting that EGFR signaling in CAFs exerted a catalytic effect on CAF-mediated cancer progression and could be regulated by the CF modification of EGFR.

In addition, the CAF-mediated EGFR signaling pathway plays promoting roles in tumor invasion and metastasis (Fig. 5). It was demonstrated that the collective invasion of SCC cells could be driven by the matrix-dependent mechano-sensitization to EGFR signaling.^258^ Because receptor tyrosine kinase (RTK) can interact exclusively with activated integrins, the ECM determines the type of RTK/integrin interaction proceeds on the cellular membrane, and this selectivity may change the intracellular location or conformation of EGFR.^99,259^ Given that the L-type calcium channel CA_V_1.1 functions with ECM stiffness and is triggered by EGFR signaling activation, calcium channel blockers may suppress SCC invasion and metastasis, and an EGFR blockade could trigger the EMT process in HNSCC.^258^ Notably, Gao et al. found that CAFs associated with high-grade serous ovarian cancer contributed to the formation of heterotypic spheroids in malignant ascites and that these CAF-centered spheroids recruited floating ovarian cancer cells, resulting in premetastatic niche formation at an early stage.^260^ In summary, these evidences support the supposition that the CAF-mediated EGFR signaling pathway is essential for several cellular functions, including maintenance of cancer stemness, cell proliferation and invasion, and metastasis. Importantly, in contrast to EGFR overexpression in tumor cells, which was positively correlated with the overall survival period of patients with several cancers,^261^ EGFR overexpression in CAFs had no significant relation to the prognosis of patients with colorectal cancer,^246^ indicating that EGFR in CAFs might not be an independent prognostic factor for survival evaluation in patients with cancers.

### Hippo signaling pathway

#### Hippo signaling pathway in CAFs and its targeted therapy

Hippo signaling pathway was originally discovered to be an important regulator of organ size in *Drosophila.*^262^ In mammals, the canonical Hippo signaling cascade consists of mammalian sterile 20-like (MST) kinases, l (LATS) kinases, and adaptor proteins Salvador homolog 1 and Mps one binder kinase activator protein. Central to this cascade signals from MST1/2 to the oncogenic of transcriptional cofactors YAP1 and its paralog transcriptional coactivator with PDZ-binding motif (TAZ).^263^ The major target transcription factors regulated by YAP/TAZ are the TEAD family.^263^ Notably, in noncanonical Hippo signaling, for instance, phosphorylated YAP1/TAZ binds directly to an angiomotin family proteins, which is in the Crumbs complex, to α-catenin, β-catenin, PTPN14, and Scribble in adherens junctions and to ZO-1/2 in tight junctions, subsequently leading to the regulation of YAP1/TAZ localization and activity independently of LATS.^264^

In contrast to other classical signal transduction pathways, such as TGF-β or Wnt signal, the Hippo pathway does not seem to be involved in special extracellular ligands or transmembrane receptors, but is regulated by upstream signals and involved in cell morphology and polarity and cell–cell and cell–ECM adhesion.^265–268^ Studying prostate cancer, Shen et al. found that the YAP1/TEAD1 protein complex transformed NFs to CAFs by activating cytoskeletal proteins and actin by regulating SRC.^47^ In addition, the proliferation of CAFs was significantly inhibited by siYAP1 or the inhibitor verteporfin,^47^ indicating that YAP1 had multiple effects on CAFs. YAP activation in CAFs^269,270^ controlled the expression of several cytoskeletal regulators, including ANLN and CTGF, and regulated actomyosin contractility and ECM remodeling in CAFs via MYL9/myosin light chain-2,^269^ and in agreement, the gain of YAP function in CAFs was associated with reactivation of actomyosin contractility and SRC,^193^ supporting the supposition that YAP/TAZ activity of CAFs was primarily associated with its effect on ECM remodeling. In another study, Calvo et al. found that YAP depletion by siRNA caused weakening of the ability of CAFs to physically contract collagen-rich matrices, and fewer focal adhesions and fewer essential structures were formed for force transmission between the cytoskeleton and matrix; however, depletion of TAZ had little effect on these processes,^269^ indicating that TAZ is not the only downstream component of the YAP-mediated signaling pathway. Interestingly, in breast cancer, the activity of the upstream negative regulators MST1/2 was not different between NFs and CAFs, while the activity of LATS kinases and phosphorylated YAP was augmented in CAFs,^269^ suggesting that YAP was not activated by the attenuated activity of the canonical MST/LATS signaling pathway in CAFs.

#### Hippo signaling pathway-mediated crosstalk of CAFs with cancer cells and its targeted therapy

CAF-mediated Hippo signaling pathway tumor-promoting activities are mainly related to cell proliferation and invasion and angiogenesis (Fig. 5). In prostate cancer, CAFs with high YAP1 expression could prompt the proliferation of cancerous epithelial cells and were more likely to cause the distant metastasis.^47^ Similarly, Shen et al. found that knocking down YAP1 or SRC in CAFs attenuated the promotion of CM on the proliferation and invasion capacity of human prostate cancer cells,^47^ indicating that CAFs in prostate cancer could promote tumor cell proliferation and invasion, which was highly dependent on the paracrine activity of YAP1 and/or SRC in CAFs. Strikingly, we found that YAP expression in primary HNSCC cells was associated with Nodal-induced metastasis; however, YAP knockdown in HNSCC cells was not associated with changes in EMT.^271^ Notably, CAF-derived miR-221 could trigger proliferative and migratory effects on MDA-MB 231 and SkBr3 breast cancer cells by interfering with the A20/c-Rel/CTGF signaling pathway.^272^ MiR-221 was reported to be strongly upregulated and closely related to the invasion and advanced clinical stages of patients with breast cancer.^273^ In general, targeting miR-221 by specific inhibitors such as LNA-i-miR-221 might cause a suppressive effect on cancer progression, especially breast tumors.^274,275^ Consistently, the ability of CAFs to promote cancer cell invasion was significantly dependent on YAP function, and loss of YAP function reduced the formation of fibrous collagen networks by CAFs and suppressed angiogenesis in vivo.^269^ In addition, in a mouse model, treatment with Y27632 (a potent, ATP-competitive inhibitor of ROCK1 and ROCK2) reduced the nuclear localization of YAP in CAFs and inhibited angiogenesis,^269^ indicating that the metastatic function of YAP in HNSCC may not be a result of EMT.

With respect to the molecular mechanism of CAF-mediated YAP/TAZ signaling pathway in the crosstalk between CAFs and cancer cells, it was reported that DKK3 in CAFs potentiated the Wnt and Hippo signaling pathways.^193^ DKK3 knockdown in CAFs decreased the levels of unphosphorylated β-catenin and TAZ, reduced nuclear YAP/TAZ translocation, and inhibited β-catenin and YAP/TAZ transcriptional activity in breast, colorectal, and ovarian cancers.^193^ DKK3 reduced YAP/TAZ degradation through the Wnt/β-catenin signaling pathway, thus acting in parallel to YAP/TAZ regulation mediated through mechanotransduction.^269,276^ Although DKK3 could activate β-catenin in CAFs, the inhibition of β-catenin by RNA interference did not affect CAF-mediated ECM remodeling or cell growth or invasion in cancer,^193^ indicating that exogenous inhibitors targeted to Wnt/β-catenin signaling axis might not attenuate aggressive behaviors of CAFs and/or that Wnt/β-catenin signaling pathway might be one of the upstream regulators of the YAP/TAZ signaling cascade. In support of this notion, DKK proteins were reported to negatively affect the surface expression of Kremen, and DKK3 was shown to inhibit Wnt signaling by triggering LRP5/6 internalization through the formation of a ternary complex with Kremen1/2 receptors.^191,192,277^ In contrast, Ferrari et al. found that Kremen1, but not LRP6, immunoprecipitated with DKK3 in CAFs, while DKK3 silencing led to Kremen1 localization to the cell periphery,^193^ demonstrating that DKK3-mediated LRP6 regulation could activate β-catenin and YAP/TAZ, with the latter being the main mediator of DKK3 functions in the crosstalk between CAFs and cancer cells.

### NF-κB signaling pathway

#### NF-κB signaling pathway in CAFs and its targeted therapy

As a ubiquitous transcription factor, NF-κB consists of five different proteins: RelA (p65), RelB, c-Rel, NF-κB1 (p50), and NF-κB2 (p52).^278^ Generally, core components of NF-κB signaling pathway are inhibitors of NF-κB (IκB) proteins, namely, the IκB kinase (IKK) complex and NF-κB transcription factor.^279,280^ In the canonical NF-κB pathway, activation is triggered by the binding of ligands (e.g., TNF-α, IL-1β) to their respective receptors (e.g., Toll-like receptors (TLRs), TNFR, and IL-1R), which leads to the phosphorylation and activation of IKK, thereof initiating the phosphorylation of IκB proteins.^281,282^ In the noncanonical NF-κB pathway, ligands such as CD40 bind to their cognate receptors. Then, this binding leads to activation of NF-κB by NF-κB-inducing kinase, which phosphorylates IKKα and facilitates IKKα phosphorylation of p100 for p52 generation. RelB/p52 heterodimers then translocate into the nucleus, subsequently leading to the activation of noncanonical NF-kB target gene expression.^283–285^

CD146, a cell membrane protein, was knocked down to promote CAFs activation, which might have been caused by the modulation of NF-κB activity.^286^ In another study, Wu et al. found that gastric cancer-derived HTRA1 promoted CAFs generation from NFs through the activation of the NF-κB/bFGF/FGF2 signaling pathway.^287^ Furthermore, CXCR2 signaling in CAFs promoted the CAF acquisition of secretory phenotype by activating NF-κB,^288^ noting that NF-κB signaling pathway was involved in regulating CAF factor secretion. Similarly, in OSCC, CAFs were stimulated with IL-1β and exhibited increased CXCL1 secretion in an NF-κB-dependent manner.^289^ Cancer-derived IL-1α increased the expression of COX-2, CXCL8, CCL20, and IL-6 in CAFs to form an inflammatory environment in pancreatic cancer.^290^ In regard to the immune response, it was reported that decreased α-SMA in CAFs was observed after their incubation with the polysaccharide MPSSS and impaired the immunosuppressive effect of CAFs through TLR4/NF-κB signaling, but there was no obvious effect on CAF viability.^291^ Ligustilide had no effect on CAFs’ viability, but reversed the immunosuppressive function of CAFs through the TLR4/NF-κB signaling pathway.^292^ In sum, these evidences suggest that NF-κB signaling pathway is mainly implicated in the activation, secretory phenotype, and immunosuppressive functions of CAFs.

#### NF-κB signaling pathway-mediated crosstalk of CAFs with cancer cells and its targeted therapy

Fundamentally, CAF-driven NF-κB was reported as a pro-inflammatory gene signature critical for tumor progression (Fig. 5). For instance, CAF-derived CXCL1, IL-6, and COX-2, known as targets of the NF-κB transcription factor, were correlated with tumor-promoting inflammation and tumor invasiveness in human breast cancer.^293^ It was reported that oxidative stress triggered NF-κB activation and STAT3 in CAFs to upregulate CCL2, and inhibition of CCL2 could reduce tumor growth of oral cancer in a mouse model.^294^ IL-6 secreted by CAFs could trigger the induction of neoplastic OPN to promote the growth, migration, and invasion of cancer cells in HNSCC via the integrin αvβ3/NF-κB pathway.^295^ Notably, in SCC, the pro-inflammatory signaling driven by CAFs was NF-κB-dependent.^36^ In sum, these data suggest that CAF-driven NF-κB signaling plays a central role in mediating protumor inflammation. Interestingly, TLR4 expressed by tumor cells was significantly associated with decreased recurrence; however, its overexpression in CAFs was independently associated with increased recurrence in patients with colorectal cancer,^296^ indicating that TLR4 in CAFs might not be an independent prognostic factor for recurrence of colorectal cancer.

In addition, CAFs could mediate the EMT and tumor stemness through a pro-inflammatory signature strictly dependent on COX-2-, NF-κB-, and HIF-1α-related signaling cascades.^297^ In pancreatic cancer, phosphorylated NF-κB was positively correlated with CAV-1 expression, and knockdown of CAV-1 in CAFs could reduce the invasiveness and motility of cancer cells, but did not affect cell proliferation.^298^ Importantly, overexpression of Smad7 in IKKβ-deficient CAFs prevented HGF secretion, and pharmacological inhibition of Met in a CAC model supported that enhanced tumor promotion was dependent on HGF-Met signaling in the mucosa of IKKβ-mutant animals,^299^ suggesting that a tumor-suppressive function of IKKβ/NF-κB in CAFs might be related to HGF release. In our preliminary study, we found that topically applied Tat-Smad7 penetrated cells in both healthy oral mucosa and oral cancer, attenuating NF-κB signaling-related inflammation.^300^ In addition, the effect of antisense oligonucleotide-miR-221 transfection in CAFs caused the inhibition of migration/invasion by downregulating NF-κB in pancreatic cancer.^301^ In addition, ablation of Saa3, a member of the serum amyloid A apolipoprotein family, in pancreatic tumor cells rendered them insensitive to the inhibitory effect of Saa3-null CAFs,^302^ suggesting that saa3 in CAFs may provide potential therapeutic benefit to pancreatic ductal adenocarcinoma (PDAC) patients. Taken together, these results suggest that CAV-1, Smad7, saa3, and miRNAs might be candidates to target the CAF-mediated NF-κB signaling pathway in cancers.

### Other signaling pathways in CAFs, crosstalk of CAFs with cancer cells, and its targeted therapy

CAF-induced signaling pathways not discussed above, including Notch,^303–305^ Hedgehog,^306–308^ p53,^309–312^ Rho/Rock,^313,314^ HIF-1α,^44,315–317^ and peroxisome proliferator-activated receptor (PPAR)^318–320^ signaling cascades, have been widely studied to understand CAFs crosstalk with cancer cells. Indeed, covering all aspects of all signaling pathways of CAFs in cancer progression is beyond the scope of this review. However, similar to the signaling pathways we summarized above, other signaling pathways exhibit unique characteristics. For instance, HIF-1α signal in CAFs promotes tumor progression mainly by regulating glycolytic metabolism. Radhakrishnan et al. demonstrated that LPA-induced glycolytic shift was the earliest, potentially priming event of the NF-CAF transition, and it was mediated through LPA/LPAR/HIF-1α signaling axis.^44^ In another example, CAF-specific RWE increased the expression of fructose bisphosphatase in cancer cells, leading to the modulation of HIF-1α function in non-small-cell lung cancer cells.^321^ Thus, all these signaling hubs in CAFs have great potential as targets for blocking CAFs crosstalk with cancer cells, and further investigations are warranted to identify the specific functions of these targets.

## Immunotheray driven by CAFs

In addition to CAFs, the TME contains an array of other nonneoplastic cells, including resident and infiltrating immune cells. Particular emphasis has been placed on T lymphocytes, tumor-associated macrophages (TAMs), myeloid-derived suppressor cells (MDSCs), and others. The immune cells in the TME play a dual role during cancer onset and progression, as they can mediate tumor clearance by killing immunogenic neoplastic cells and contribute to tumor escape by shaping tumor immunogenicity.^322,323^ Immunotherapies, harnessing the dual roles of the immune system in battling cancer, have emerged as new pillars within oncotherapy.^324,325^

To date, CAFs have been found mainly to regulate and rewire the TME to favor the malignant biological properties of tumors through the interaction of CAFs and T lymphocytes. In colorectal cancer, CD70 expression in CAFs and the CD70/CD27 axis affected the expansion and differentiation of T lymphocytes by activating the NF-κB signaling pathway.^326^ Chakravarthy et al. found that pan-cancer ECM dysregulation was linked to CAF-mediated TGF-β signaling, which was closely related to immunological activity and could predict failure of PD-1 blockade.^327^ Meanwhile, the combination of TGF-β blockers and anti-PD-L1 antibodies promoted T cell penetration into the tumor center, improving tumor responsiveness to anti-PD-L1 therapy.^328^ Activated CAFs can also produce TGF-β to form a positive-feedback loop for CAFs activation. Therefore, inhibition of TGF-β signaling, such as through treatment with galunisertib (LY2157299) or AVID200, is worthy of further exploration as a method to improve PD-L1 immunotherapy.^329–332^ In addition to TGF-β blockers, pirfenidone (PFD)-targeting CAFs possessed inhibitory effects on migration and decreased the expression of PD-L1 by targeting CCL17 and TNF-β. The regulation of CAFs through PFD treatment may reduce the acquisition of CAF-mediated immunosuppressive capacity in breast cancer cells, thereby leading to increased efficacy of chemotherapy.^333^ In addition, highly expressed presenilin 1 in CAFs also regulated tumor-infiltrating lymphocyte populations in the TME via the Wnt/β-catenin pathway. Inhibition of presenilin 1 by IL-1β promoted the proliferation and penetration of CD8^+^ T cells in multiple ovarian models and retrieved functional CD8^+^ T cells in the TME, which may improve the efficacy of current immunotherapies.^334^ The angiotensin receptor blocker losartan can drive myofibroblast CAFs into a quiescent state, attenuate immunosuppression, and increase T lymphocyte activity, thereby significantly improving the response of breast cancer cells to immune checkpoint blockers.^335^ Feig et al. found that pancreatic cancer cells coated with the CAF-derived CXCL12 caused the depletion of T cells and contributed to cancer unresponsiveness to α-PD-L1; administering a CXCR4 (a CXCL12 receptor) inhibitor, AMD3100, blocked CAF-directed immune evasion of the tumor to increase T cell infiltration in cancer cells and greatly diminish cancer volume when administered in combination with α-PD-L1.^336^ These studies demonstrate that the dysregulation of CAFs contributes to tumor-induced immunosuppression and that immunotherapy driven by CAFs sensitizes tumors to T cells, stimulating strong antitumor cellular immunity and tumor regression.

In addition, immunotherapies targeting TAMs have drawn significant attention, as they deplete and/or reprogram TAMs to block their protumor functions or restore their tumoricidal properties.^337^ Colony-stimulating factor-1 receptor (CSF1R) inhibitors targeting TAMs successfully in diffuse-type giant cell tumors failed to deliver an antitumor effect in other tumor models, as CSF1R inhibition altered CXCL1 and other chemokine secretion by CAFs and triggered a profound increase in protumor polymorphonuclear myeloid-derived suppressor cell (PMN-MDSC) recruitment to tumors; combined inhibition of CSF1R and CXCR2 (a CXCL1 receptor on granulocytes) blocked CAF-mediated MDSC recruitment and reduced tumor growth.^338^ In OSCC, CAFs educated macrophages to suppress T cell proliferation, which was restored by the neutralization of TGF-β, IL-10, or arginase I.^339^ The CAF–TAM interaction was also regulated by pleiotropic glycoprotein stanniocalcin-1, which was secreted by CAFs and partially suppressed TAM differentiation.^340^ In colorectal cancer, CAFs could attract monocytes via IL-8/CXCR2 pathway to induce their polarization.^341^ Thus, CAFs play crucial roles in shaping the immunosuppressive TME by educating TAMs to induce a protumor phenotype, and methods for reversing CAF-mediated immunosuppression in TAM-targeted therapeutics need to be considered. In addition, CAFs can functionally sculpt other immune cells in the TME through their high secretory ability. CAF-derived cytokines, including IL-6 and CXCL12, induced the generation and activation of MDSCs to favor HCC progression.^342^ HCC CAFs recruited normal dendritic cells and educated them to acquire a tolerogenic phenotype through IL-6/STAT3 signaling.^343^ Taking these observations together, it can be concluded that crosstalk between CAFs and T cells, TAMs, MDSCs, etc. is involved in the formation of tumor immunosuppression (Fig. 5), and combination therapy driven by CAFs and immunotherapies might be an effective and promising strategy for treating insensitive tumors.

Meanwhile, the heterogeneity of intratumoral CAFs impels a paradigm shift in CAFs behaviors, especially immune-related function. Although the classification criteria for CAF subtype are diverse and are in a state of flux, some subpopulations of CAFs with an immunomodulating phenotype likely make more distinct contributions to tumor immunity. Based on the cellular source, Bartoschek et al. found that mCAFs from resident fibroblast can also regulate the immune response.^57^ Unlike this, from the perspective of function, in PDAC, iCAFs revealing a pro-inflammatory gene signature with IL-6 and CXCL12 expression promoted tumor growth, angiogenesis, and macrophage recruitment.^344^ Similarly, in breast cancer, CAF-S1 fibroblasts attracted or regulated the function and differentiation of T lymphocytes by secreting CXCL12 to format an immunosuppressive environment.^58^ Further, the CAF subtypes with a high level of IL-6 and FAP, which associated with a large cluster of pathways involved in immune regulation and the worse survival outcomes in ovarian cancer.^345^ These data suggest that the classification of different subpopulations based on CAFs’ function seems to be much more reliable in tumor immunity.

## Therapeutic resistance caused by CAFs

At present, a number of CAF-mediated anticancer therapies have been reported, of which most are in phases of preclinical trials. Overall, CAF-mediated anticancer therapies mainly include the following five application approaches: inhibiting transformation from normal fibroblasts to CAF, promoting transformation from CAF to normal fibroblasts, inhibiting tumor development and progression, activating immunity system, and reversing tumor chemoresistance (Table 4). We found that breast cancer is the most widely studied for targeting CAFs in cancer treatment. Interestingly, no trials of pancreas cancer were found in the inhibition of tumor development and progression; however, a majority of studies in reversing tumor chemoresistance were related to pancreas cancer, suggesting that CAF-mediated chemoresistance might be tumor- or organ-specific. The interaction between tumor cells and CAFs or ECM blunts the response to cancer cell-directed therapies, including chemotherapy, small-molecule drug-based therapy, and radiotherapy (RT).^346^

**Table 4:** CAF-mediated anticancer therapies

Target	Cancer cell origin	Study type (sample size)	Anticancer therapies (NCT number)	Anticancer mechanisms	Refs.
Inhibiting transformation from normal fibroblasts to CAF					
NOX4	Head, neck, colorectum	Preclinical	GKT137831	Delays transdifferentiation of fibroblast to CAF	^530^
TGF-β	Breast, ovary	Preclinical	HA-PTX/MATT HNPs; LY2157299	Blocks the fibroblast activation by downregulating the TGF-β expression	^531,532^
Promoting transformation from CAF to normal fibroblasts					
CAF	Solid tumors	Preclinical	sTRAIL LPD	Induces fibroblast reprogramming	^533^
TGF-β	Breast	Preclinical	Artemisinin derivatives	Inactivates CAF and inhibits metastasis	^534^
VDR	Pancreas	Preclinical	Calcipotriol	Reprises the quiescent state of CAF	^535^
Vitamin A	Pancreas	Preclinical	ATRA	Induces quiescence of stellate cells	^536^
Inhibiting tumor development and progression					
CAF	Esophagus, stomach	Preclinical	Bortezomib + panobinostat	Reduces the viability of CAF through inducing caspase-3-mediated apoptosis	^537^
CAF	Lung	Preclinical	5-Fluorouracil	Eliminates CAF recruited by tumors	^538^
CAF	Stomach	Preclinical	Losartan + others	Reduces CAF activity and collagen fiber	^539^
CSF1R + CXCR2	Lung, breast, colon, others	Preclinical	JNJ-40346527 + SB225002	Blocks protumor PMN-MDSC recruitment and reduces tumor growth	^338^
FAP	Esophagus, breast, lung, colon, rectum	Preclinical; phase I (43)	PIT; ^131^I-mAbF19 PDT; Sibrotuzumab; αFAP-PE38	Depletes CAF specifically or inhibits function of CAF to suppress tumor progression	^382,540–545^
FAP + DPPIV	Lung	Preclinical	FAP deletion; PT630	Increases collagen level, decreases CAF content and blood vessel density in tumor	^546^
Integrin αvβ3	Breast	Preclinical	ProAgio	Reduces intratumoral collagen and decreases growth factors from CAF	^547^
MMP	Breast, solid tumors	Preclinical; phase I (32)	HA-PTX/MATT HNPs; S-3304	Suppresses angiogenesis, degradation of extracellular matrix, and metastasis	^531,548^
MiR-214 + miR-301a	Stomach	Preclinical	Astragaloside IV	Reduces expression of oncogenic pluripotency factors SOX2 and NANOG	^549^
PDGFR signaling	Cervix uteri	Preclinical	Imatinib	Reduces epithelial cell growth factor and angiogenic factor by CAF	^550^
ROS/MAPK + ferroptosis pathways	Nasopharynx	Preclinical	Disulfiram/copper	Induces cytotoxic effects on CAF and tumor cells, promotes CAF apoptosis, and inactivates CAF	^179^
TEM-1	Colorectum	Phase II (126)	MORAb-004 (NCT01507545)	Blocks specific TEM-1 receptor–ligand interactions	^551^
Tenascin	Brain	Phase II (43)	^131^I-m81C6	Delays tumors growth and metastasis	^552^
TGF-β	Breast, liver	Preclinical	Pirfenidone; LY2109761	Induces CAF apoptosis and inhibits CAF proliferation	^90,553^
VEGF	Pleura	Phase III (448)	Bevacizumab + others	Inhibits tumor angiogenesis	^554^
VDR/1RARβ	Skin	Preclinical	EB1089; LE135	Reduces cancer cell proliferation and/or increases apoptosis	^318^
Activating immunity system					
CAF	Liver	Preclinical	DC/CAF fusion	Activates cytotoxic T lymphocytes	^555^
CCR2 + IDO1 + NOX2	Lung	Preclinical	CCR2i + epacadostat + GSK-2795039	Reverses the interaction between CAF and monocytic myeloid cells	^556^
CXCR4	Pancreas	Preclinical	AMD3100 + others	Reverses tumor immune evasion	^336^
FAP	Colon, breast	Preclinical	DNA vaccine; PIT	Enhances cytotoxic T cell infiltration	^336,545^
PD-L1 + TGF-β	Breast, colorectum	Preclinical	M7824	Activates innate and adaptive immune to suppress tumor growth and metastasis	^557^
Reversing tumor chemoresistance					
AR	Pancreas	Phase II (49)	Losartan + others	Reduces collagen and hyaluronan level	^558^
ATRA	Pancreas	Preclinical	ATRA + gemcitabine	Targets multiple tumor–stromal pathways	^559^
Collagen I	Breast	Preclinical	Losartan-loaded C_16_-N	Inhibits CAF and collagen synthesis	^560^
CTGF	Pancreas	Preclinical; phase I (50)	FG-3019 gemcitabin (NCT01181245)	Decreases X-linked inhibitor of apoptosis protein to kill tumor cell	^561^
FAP-α	Prostate	Preclinical	Drug-loaded CAP-NPs	Disrupts stromal barrier to drug	^562^
GPR77	Breast	Preclinical	Anti-GPR77 antibody	Reduces the infiltration of CAF	^563^
Hedgehog pathway	Pancreas	Preclinical	IPI-926 + gemcitabine	Depletes CAF to increase intratumoral concentration of gemcitabine	^395^
Hyaluronan	Pancreas	Phase II (279)	PEGPH20 + others	Increases drug delivery	^564^
IL-6	Stomach	Preclinical	Tocilizumab	Renews chemotherapy-induced apoptosis	^388^
mTOR/4E-BP1 pathway	Pancreas	Preclinical	SOM230 + gemcitabine	Blocks insoluble or soluble proteins synthesis and secretion from CAF	^565^
Vimentin + SMA	Pancreas	Phase I (7)	PEGPH20 + Avelumab (NCT03481920)	Increases drug delivery and immune infiltration	^566^

*4E-BP1* 4E-binding protein 1, *AR* angiotensin receptor, *ATRA* all-*trans* retinoic acid, *CAF* cancer-associated fibroblast, *CAP-NP* cleavable amphiphilic peptide nanoparticles, *CSF1R* colony-stimulating factor-1 receptor, *CTGF* connective tissue growth factor, *CXCR2* chemokine receptor 2, *DC* dendritic cells, *DPPIV* dipeptidyl peptidase IV, *ECM* extracellular matrix, *FAP* fibroblast activation protein, *GPR77* G-protein-coupled receptor 77, *HA-PTX/MATT* hyaluronic acid-paclitaxel/marimastat, *HNP* hybrid nanoparticles, *IL* interleukin, *LPD* lipid-coated protamine DNA complexes, *MAPK* mitogen-activated protein kinase, *MMP* matrix metalloproteinases, *mTOR* mammalian target of rapamycin, *NOX4* NAD(P)H oxidase-4, *PEGPH20* pegvorhyaluronidase alfa, *PDGFR* platelet-derived growth factor receptor, *PDT* photodynamic therapy, *PIT* photoimmunotherapy, *PMN-MDSC* polymorphonuclear myeloid-derived suppressor cells, *RAR* retinoic acid receptor, *ROS* reactive oxygen species, *SMA* smooth muscle actin, *sTRAIL* secretable TNF-related apoptosis-inducing ligand, *TGF* transforming growth factor, *TEM-1* tumor endothelial marker-1, *VEGF* vascular endothelial growth factor, *VDR* vitamin D receptor.

**Fig. 6:**
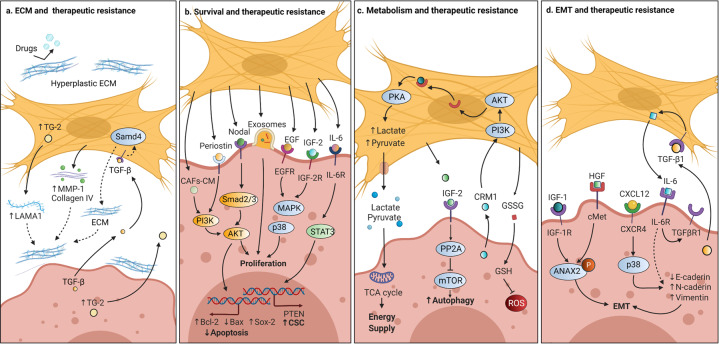
CAF-mediated therapeutic resistance in anticancer treatment. **a** CAFs secreted dense desmoplastic matrix, including laminin A1, type IV collagen into the TME hindering the delivery of drugs, and increasing the resistance. **b** CAFs released regulatory molecules such as periostin, Nodal, EGF, IGF-2, and IL-6 or exosomes, which activated pro-survival signaling pathways, including proliferation, stemness, and apoptosis of cancer cells enhancing therapeutic resistance. **c** CAFs fed increased pyruvate, lactate, or GSSG to tumor cells by increasing energy supply and decreasing ROS. It also activated IGF-2 to increase autophagy via downstream pathways and the changes of metabolism in CAFs and tumor cells maintain the tumor survival in treatment. **d** CAFs released regulatory factors, including IGF-1, HGF, CXCL12, IL-6, and TGF-β1, to mediate EMT process through downstream ANAX2 and p38 pathways, subsequently increasing metastasis and resistance. TG2 tissue transglutaminase 2, LAMA1 laminin A1, Bcl-2 B cell lymphoma 2, Bax Bcl-2-associated X protein, Sox-2 SRY (sex-determining region Y)-box 2, TCA cycle tricarboxylic acid cycle, PP2A protein phosphatase 2A, CRM1 chromosomal region maintenance 1, GSSG glutathione disulfide, GSH glutathione, ANXA2 annexin A2

Chemotherapy utilizing traditional cytotoxic antitumor drugs plays therapeutic roles in the lung, pancreatic, colorectal cancer, and other malignant tumors.^347–350^ First, CAFs generate physical obstacles around malignant elements by secreting a dense desmoplastic matrix into the TME. Laminin A1 secreted by CAFs, as well as tissue transglutaminase produced by PDAC cells, altered the response to gemcitabine,^351^ and breast CAFs increased MMP1 secretion synergistically with type IV collagen to promote Taxotere resistance via the TGF-β signaling, while GM6001 (an MMP1 inhibitor) recovered cancer cell chemosensitivity,^352^ perhaps by establishing physical barriers or inducing microvasculature compression through CAFs, and overcoming CAF-driven ECM hindrance of chemotherapeutic drugs delivery. Then, CAFs released regulatory molecules into tumor tissue in a paracrine-^353–356^ or exosome-driven^357^ manner to protect tumor cells from being eliminated. CAFs that were cocultured with cancer cells secreted IL-6 and upregulated the expression of CXCR7 through the STAT3/NF-κB pathway, which enhanced the proliferation and resistance of ESCC to cisplatin.^218^ In HNSCC, CAFs can upregulate autophagy by increasing the secretion of IL-6 and IL-8, thereby reducing cell sensitivity to cisplatin.^358^ In addition, CAFs can promote tumor stemness through the secretion of IL-17a and POSTN.^190,359,360^ Together, these data suggest that CAFs stimulated by agents in chemotherapy activate pro-survival signaling pathways, including those promoting the proliferation, stemness, and autophagy of cancer cells, to enhance treatment resistance (Fig. 6). In addition, activated CAFs can reprogram tumor cell metabolism to maintain tumor cell survival and protect them from apoptosis induced by treatment and/or stress. Augmented pyruvate in CAFs fed to tumor cells increased the activity of the tricarboxylic acid cycle to promote the survival of lymphoma cells.^361^ EMT mediated by CAFs decreased cellular adhesion, which was beneficial to tumor metastasis and resistance.^362^

Small-molecule drugs targeting signaling pathways for tumor progression and growth have received attention and are widely used in the treatment of lung, breast, head and neck, and liver cancer.^363–365^ In comparing the potential mechanisms of CAF-mediated drug resistance that are substantially similar to those of chemical resistance, certain aspects of CAF-mediated drug resistance are unique (Fig. 6). To respond to drugs, the tumor–stroma or TME targets specific pathways and attenuates the effects of the drugs. For instance, excess CAF-secreted EGF can competitively bind to EGFR with cetuximab and activate MAPK to promote cetuximab resistance in colorectal cancer.^253^ Similarly, expressed PDGF-C in CAFs promotes angiogenesis and inhibits the effect of VEGF inhibitor.^366^

Similar to chemotherapy, CAF-induced pro-survival signaling, including proliferation, autophagy, and stemness, is able to induce resistance to RT (Fig. 6).^367–369^ In addition, the formation of a hypoxic microenvironment and the EMT driven by CAFs can also confer radioresistance onto cancer cells.^370–373^ In particular, we concluded that CSCs played an important role in the poor efficacy of RT and that CSCs survived more easily due to their potential innate radioresistance, which was partially induced by CAFs.^374^ CAFs induced a reduction response to antitumor treatment by maintaining activity via an autocrine periostin loop even during RT,^371^ and caused radiation-induced fibrosis, which was associated with retinoic acid or TGF-β,^375^ and with a severe adverse response to RT.^376^ These properties of CAFs make them potential targets for sensitizing tumors exposed to RT.

## Challenges and future perspectives

In the present review, we concluded that several CAF-mediated signaling pathways exerted a supportive role in cancer progression. Key signaling pathway components, biomarkers in CAFs and CAF-derived factors, miRNAs, lncRNAs, etc., were predicted or found to have great potential for targeted therapy. Importantly, several clinical trials on CAFs have also been performed and shown that CAFs have a promising future in cancer therapy (Table 5). However, there are also multiple hurdles that need to be overcome before targeting CAFs in cancer treatment.

**Table 5:** CAF-directed therapeutic resistance

Therapy	Cancer type	Key molecule from CAFs	Molecular mechanism in cancer cell	Refs.
Chemotherapeutics				
5-FU	GC	Paracrine low SPARC	Activates AKT/mTOR and MEK/ERK pathway	^148^
Adriamycin	PRAD	Paracrine IL-6 and exosomal miR-423	Activates JAK/STAT and TGF-β pathway, and upregulates glutathione and GREM2	^419,451,567^
Paclitaxel				
Adriamycin	BC	Paracrine CXCL12/HMGB1	Downregulates H2AX phosphorylation	^568,569^
Adriamycin	SC	Paracrine Nodal	Activates Nodal/Samd/AKT pathway	^139^
Cisplatin	ESCA	Paracrine PAI-1/IL-6	Activates AKT and ERK1/2 and CXCR7 pathway	^218,523,570^
Cisplatin	LC	Paracrine SDF-1/ANXA3/IL-6	Activates NF-κB/Bcl-2 and JNK pathway, and upregulates p53	^219,312,399,571^
Cisplatin	HNSCC	Exosomal miR-196a	Upregulates CDKN1B, ING5 LC3-II, and Beclin-1	^435,572^
Cisplatin	OC	Paracrine POSTN/CXCL12 and exosomal miR-98	Activates STAT3, PI3K/AKT, and /Wnt/β-catenin pathway and downregulates CDKN1A	^440,467,525,573^
Cisplatin	LUAD	Paracrine IL-11/COX-2	Activates IL-11/STAT3 pathway and downregulates TNFSF4	^461,529,574^
Cisplatin	HCC	Paracrine HGF	Activates c-Met and Mec-ERK1/2 pathway	^487^
Cisplatin	VSCC	Exosomal lncRNA UCA1	Activates miR-103a/WEE1 pathway	^575^
Docetaxel	BC	Paracrine IL-8	Upregulates CXCL2, MMP1, IL-8, RARRES1, FGF1, and CXCR7	^362,576^
Ebimycin	BC	Paracrine pyruvate and lactate	Upregulates mitochondrial activity	^577^
Gemcitabine	PDAC	Paracrine LAMA1/Survivin/IL-6/SDF-1/MMP3/MMP9/PDGF/and CCL-7 and exosomal Snail/miR-146a/miR-106b	Activates protein kinase, AKT, and SDF-1/CXCR4/SATB-1 pathway, and upregulates Snail and TP53INP1	^70,309,351,433,478,565,577^
Oxaliplatin	HNSCC	Paracrine IL-6 and IL-8	Activates autophagy pathway	^358^
Oxaliplatin	CRC	Paracrine CM from CAFs	Activates STAT3 and p38 pathway	^578^
5-FU				
Paclitaxel	BC	Paracrine MMP1 and collagen IV	Activates TGF-β pathway	^352^
Paclitaxel	LUAD	Paracrine HGF	Activates c-Met/PI3K/AKT pathway	^579^
Paclitaxel	OC	Paracrine CM from CAFs	Upregulates LPP	^580^
Targeted therapeutic				
Cetuximab	CRC	Paracrine EGF	Activates MAPK pathway	^253^
Cetuximab	HNSCC	Paracrine-soluble factors	Upregulates MMP1	^319,581^
Lenzclutamiad	PRAD	Paracrine CM from CAFs	Activates PI3K/AKT pathway and upregulates E-cadherin and vimentin	^582^
EGFR-TKI	LUAD	Paracrine HGF	ND	^255^
Gefitinib	LC	Paracrine CM from CAFs	Activates AKT and ERK, ANXA2/EMT, and hedgehog pathway	^489,583,584^
Erlotinib				
Trastuzumab	BC	Paracrine IL-6/FGF-5	Activates NF-κB, JAK/STAT3, AKT, and c-SRC/HER2 pathway	^585,586^
Trastuzumab	BC	Paracrine pyruvate/lactate/ fibronectin	Activates integrin-β1 pathway and promote mitochondrial activity	^577,586,587^
Tamoxifen				
Sorafenib	PRAD	Paracrine CM from CAFs	Activates autophagy pathway and upregulate AKT phosphorylation and Bcl-xL	^588^
Sorafenib	HCC	Paracrine HGF	Activates c-Met and Mec-ERK1/2 pathway	^487^
Radiotherapy therapy (RT)				
RT	LC	Paracrine FGF/IGF-2	Activates autophagy pathway	^369,589^
RT	PDAC	Paracrine-soluble factors	Activates protein kinase and AKT pathways	^590^
RT	CESC	Paracrine IGF-2, EGF, FGF-4, IGFBPs, and GM-CSF	Activates p38 pathway	^591^
RT	LUAD	Paracrine CXCL12	Activates CXCL12/CXCR4 pathway	^591^
RT	ESCA	Paracrine PDGFβ	Activates PDGFβ/PDGFβR/FOXO1 pathway and upregulates lncRNA DNM3OS	^592^
RT	CRC	Exosomal TGF-β/IGF-1	Activates TGF-β and IGF-1/IGF1R pathway	^368,421,490^

*ANXA3* annexin A3, *AKT* protein kinase B, *B-ALL* B cell acute lymphoblastic leukemia. *BC* breast cancer, *CESC* cervical and endocervical cancer, *CM* conditioned medium, *COX-2* cyclooxygenase, *CRC* colorectal cancer, *EGF* epidermal growth factor, *ERK* extracellular signal-related kinase, *ESCA* esophageal carcinoma, *FGF* fibroblast growth factor, *GC* gastric cancer, *HCC* hepatocellular carcinoma, *HGF* hepatocyte growth factor, *HMGB1* high-mobility group box 1, *HNSCC* head and neck squamous cell carcinoma, *IGF* insulin-like growth factor, *IL* interleukin, *JAK* Janus kinase, *LAMA1* laminin subunit alpha 1, *LC l*ung cancer, *LUAD* lung adenocarcinoma, *MMP* matrix metalloproteinases, *mTOR* mammalian target of rapamycin, *ND* not determined, *NF-κB* nuclear factor kappa-B, *OC* ovarian cancer, *PDAC* pancreatic ductal adenocarcinoma, *PAI-1* plasminogen activator inhibitor 1, *PDGF* platelet-derived growth factor, *PI3K* phosphatidylinositol-3-kinase, *POSTN* periostin, *PRAD* prostate adenocarcinoma, *RCC* renal cell carcinoma, *SC* stomach cancer, *SPARC* secreted protein acidic and rich in cysteine, *STAT* signal transducer and activator of transcription, *TGF* transforming growth factor, *TSCC* tongue squamous cell carcinoma.

**Fig. 7:**
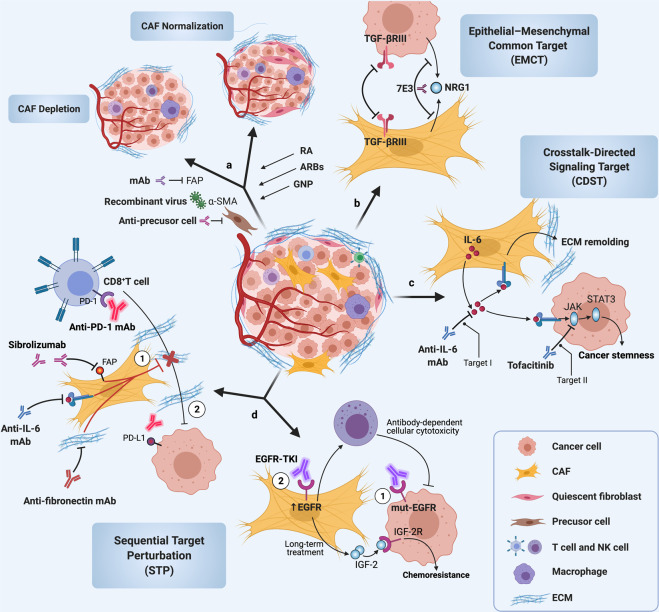
CAF-driven targeted therapies and alternative targeting avenues, including epithelial–mesenchymal common target (EMCT), sequential target perturbation (STP), and crosstalk-directed signaling target (CDST) for CAF-directed or host cell-directed antitumor therapy. **a** Besides the inhibition of NFs to CAFs, there are still two major ways to decrease the CAFs number in TME: (i) targeting specific markers of CAFs by monoclonal antibody (mAb), recombinant virus, anti-precursor cell to deplete CAFs; (ii) revert the activated CAFs to quiescent phenotype by retinoic acid (RA), angiotensin receptor blockers (ARBs), gold nanoparticle (GNP), etc. **b** Both simultaneous overexpression of the same molecular protein in CAFs and cancer cells like TGF-βRIII and NRG1 has the potential to be targeted as EMCT. **c** In the regard to CAF-mediated signaling pathways in crosstalk with cancer cells, CDST is targeting simultaneously two different components of one signaling cascade in cancer cells and CAFs, respectively, in which the trigger locating in the upstream of CAFs (Target I), while effector locating in the downstream of cancer cells (Target II). **d** EGFR-TKI targets the mut-EGFR in cancer cells, while EGFR is up-expressed in CAFs. STP on cancer cells and CAFs, targeting the EGFR in cancer cells firstly (Step①), then in CAFs (Step②), may promote NK cell function to enhance antitumor efficacy and also avoid the IGF-2-mediated chemoresistance after long-term treatment. Since there are different functions in cancer cells and CAFs, STP also aims at targeting CAFs to block the protumor effect firstly (Step①: such as targeting ECM remodeling to move the barrier for CD8^+^ T cell infiltration) and then aiming at cancer cells (Step②: such as performing PD-1/PD-L1 to antitumor immunotherapy) might obtain well therapeutic efficacy

First, how would decreasing the number of CAFs directly attenuate their tumor-promoting effects? Ample evidence suggests that FAP is an excellent target for CAFs, and antibodies against FAP and other FAP-targeting drugs are in development.^377^ T cell-mediated CAFs could be depleted by a DNA vaccine targeting FAP.^378,379^ Although FAP-specific CAR-T cells can selectively kill CAFs, they have been found to cause extensive lethal osteotoxicity and cachexia due to FAP expression in MSCs.^338,380,381^ In this regard, nanoparticles based on ZnF16pc loading and FAP-specific single-chain variable fragments or αFAP-Z@FRT can target FAP to effectively and safely deplete CAFs.^382^ In addition to CAF depletion, sibrolizumab (an anti-FAP monoclonal antibody) has been shown to target and prevent the activation of FAP^+^ CAFs to inhibit tumor growth.^383,384^ From another perspective, Noggin (a BMP signaling pathway inhibitor) can reverse the pericyte-CAF transition to decrease the number of CAFs.^127^ Notably, in addition to CAF depletion, the prevention of CAF generation and CAF normalization highly depend on their cellular phenotypes (Fig. 7a). Thus, there is an urgent need to enhance the understanding of the heterogeneity of CAFs and find more specific therapeutic targets.

Second, since targeting cancer cells can unexpectedly render a therapeutic failure or even an acceleration of cancer, the option of EMCT targeting both in cancer cells and their adjacent stromal cells have become an attractive alternative. We have previously demonstrated that the common target perturbation of TGF-βRIII in oral cancerous epithelial cells and CAFs in conjunction with multiple antitumor effects that include depression of angiogenesis and metastasis and promotion of CAFs transition to an NF type, attenuating the CAF support of tumors.^385^ This finding suggests that the candidates for EMCTs are based on their simultaneous overexpression (Fig. 7b). Indeed, differentially expressed proteins in cancer cells and CAFs are common events in tumor progression. For these cases, we propose an alternative approach: sequential target perturbation (STP) in cancer cells and CAFs might lead to therapeutic efficacy, with targeting of CAFs to block the protumor effect first and then treating cancer cells to realize anticancer effects (Fig. 7d).

Third, to date, targeting two components of one signaling pathway related to specific tumor biology in cancer cells has been performed to treat malignancies.^386,387^ Since the crosstalk between cancer cells and CAFs is mediated by complexed signaling networks, we propose a novel therapeutic approach involving crosstalk-directed signaling targets (CDSTs) that simultaneously target two different components of one signaling cascade in cancer cells and CAFs, in which the trigger is located upstream of the CAFs and the effector is located in the downstream of the cancer cells (Fig. 7c). For instance, as CAF-derived IL-6 is beneficial for ECM remodeling^218,219^ and CAF generation^220^ to fuel tumor progression, and because IL-6 activates the IL-6/JAK/STAT3 signaling pathway in cancer cells,^236,237^ combining an IL-6-neutralizing antibody^236^ to treat the CAFs and the JAK inhibitor tofacitinib to treat the cancer cells^388,389^ might be valuable to study the targeted therapy. CAFs and their related signaling and/or downstream effectors in cancer cells are underexplored targets for cancer therapy.

Fourth, immunotherapy is considered an established pillar of cancer treatment; however, the current questions to answer include why does immunotherapy work well in some cancers but not at all in others? One of the major reasons that cancers fail to respond to anti-PD-1/PD-L immune checkpoint therapy is that CD8^+^ T cells cannot infiltrate into the TME of “Cold Tumor.”^390^ An important reason for this failure is CAF-mediated ECM forming a physical barrier to prevent CD8^+^ T cell infiltration and limits the delivery of drugs.^391,392^ Currently, there are three strategies for targeting CAF-mediated ECM remodeling: targeting the producer of ECM (activated CAFs),^393^ targeting the signaling pathways for ECM remodeling,^394,395^ and targeting ECM components.^396,397^ In this regard, with a method similar to STP, we propose that targeting CAF-mediated ECM to move the barrier first and then performing cancer immunotherapy might optimize therapeutic efficacy. In addition, since CAFs can recruit the PMN-MDSCs for protumor progression and resistance, attenuating this recruitment is a potential future cancer treatment. In sum, promising CAF-driven immunotherapy might focus on ECM normalization, prevention of disturbances from non-therapeutic immune cells, and combining other antitumor therapies with immunotherapy to maximize the efficacy of cancer immunotherapy.

In conclusion, since CAF-mediated signaling pathways have been implicated in the crosstalk with cancer cells that promote tumor progression and can affect the antitumor therapeutic efficacy, considering the evidence presented in this review, our group hypothesizes that EMCTs, STP, and CDSTs are alternative targeting avenues for CAF- or host cell-directed antitumor therapy.
